# ZEB1‐regulated inflammatory phenotype in breast cancer cells

**DOI:** 10.1002/1878-0261.12098

**Published:** 2017-07-11

**Authors:** Akihiro Katsura, Yusuke Tamura, Satoshi Hokari, Mayumi Harada, Masato Morikawa, Tsubasa Sakurai, Kei Takahashi, Anna Mizutani, Jun Nishida, Yuichiro Yokoyama, Yasuyuki Morishita, Takashi Murakami, Shogo Ehata, Kohei Miyazono, Daizo Koinuma

**Affiliations:** ^1^ Department of Molecular Pathology Graduate School of Medicine The University of Tokyo Japan; ^2^ Department of Respiratory Medicine and Infectious Disease Graduate School of Medical and Dental Sciences Niigata University Japan; ^3^ Department of Metabolic Care and Endocrine Surgery Graduate School of Medicine The University of Tokyo Japan; ^4^ Department of Microbiology Faculty of Medicine Saitama Medical University Moroyama Japan; ^5^ Present address: Division of Molecular Biotherapy Cancer Chemotherapy Center Japanese Foundation for Cancer Research Tokyo 135‐8550 Japan

**Keywords:** IL‐6, IL‐8, TGF‐β, ZEB1, ZEB2

## Abstract

Zinc finger E‐box binding protein 1 (ZEB1) and ZEB2 induce epithelial‐mesenchymal transition (EMT) and enhance cancer progression. However, the global view of transcriptional regulation by ZEB1 and ZEB2 is yet to be elucidated. Here, we identified a ZEB1‐regulated inflammatory phenotype in breast cancer cells using chromatin immunoprecipitation sequencing and RNA sequencing, followed by gene set enrichment analysis (GSEA) of ZEB1‐bound genes. Knockdown of ZEB1 and/or ZEB2 resulted in the downregulation of genes encoding inflammatory cytokines related to poor prognosis in patients with cancer, including *IL6* and *IL8*, therefore suggesting that ZEB1 and ZEB2 have similar functions in terms of the regulation of production of inflammatory cytokines. Antibody array and ELISA experiments confirmed that ZEB1 controlled the production of the IL‐6 and IL‐8 proteins. The secretory proteins regulated by ZEB1 enhanced breast cancer cell proliferation and tumor growth. ZEB1 expression in breast cancer cells also affected the growth of fibroblasts in cell culture, and the accumulation of myeloid‐derived suppressor cells in tumors *in vivo*. These findings provide insight into the role of ZEB1 in the progression of cancer, mediated by inflammatory cytokines, along with the initiation of EMT.

**Abbreviations**
ATCC: American Type Culture Collection
BM: bone marrow
CCL: CC *chemokine* ligand
CCLE: Cancer Cell Line Encyclopedia
CD: cluster of differentiation
CDH1: cadherin‐1
ChIP‐seq: chromatin immunoprecipitation sequencing
CSF2 (GM‐CSF): granulocyte–macrophage colony‐stimulating factor
CSF3 (G‐CSF): granulocyte colony‐stimulating factor
CXCL: chemokine (C‐X‐C motif) ligand
DMEM: Dulbecco's modified Eagle's medium
EDTA: ethylenediaminetetraacetic acid
ELISA: enzyme‐linked immunosorbent assay
EMT: epithelial‐mesenchymal transition
ER: estrogen receptor
ESRP: epithelial splicing regulatory protein
FBS: fetal bovine serum
FPKM: fragments per kilobase of exon per million mapped reads
FSC: forward scatter
GFP: green fluorescent protein
GSEA: gene set enrichment analysis
HBSS: Hanks’ balanced salt solution
HEPES: 4‐(2‐hydroxyethyl)‐1‐piperazineethanesulfonic acid
HER2: human epidermal growth factor receptor 2
ICAM‐1: intercellular adhesion molecule 1
IDO: indoleamine 2,3‐dioxygenase
IL: interleukin
JCRB: Japanese Collection of Research Bioresources
MDSC: myeloid‐derived suppressor cell
Mo: monocytic
NEAA: nonessential amino acids
NES: normalized enrichment score
PAI‐1: plasminogen activator inhibitor‐1
PLAUR: plasminogen activator, urokinase receptor
PMN: polymorphonuclear
PR: progesterone receptor
RNA‐seq: RNA sequencing
RPMI: Roswell Park Memorial Institute
S.D.: standard deviation
SDS: sodium dodecyl sulfate
STAT: signal transducer and activator of transcription
TGF‐β: transforming growth factor‐β
ZEB: zinc finger E‐box binding protein

## 1 Introduction

Breast cancer is a leading cause of cancer death in female patients. It is a heterogeneous disease, similar to cancers of other organs (Badve *et al*., 2011), and it is an especially troubling disease because healthy young women without any previous history of disease are often affected and show poor prognosis (Anders *et al*., 2008). In particular, triple‐negative breast cancers that do not express the estrogen receptor (ER), progesterone receptor (PR), and human epidermal growth factor receptor 2 (HER2) are associated with very poor prognosis because they have a strong ability to metastasize, a high risk for relapse, and are refractory to chemotherapy (Anders and Carey, 2009). Among several subtypes of breast cancer, basal‐type breast cancer accounts for most triple‐negative breast cancers and shows a mesenchymal phenotype that is accompanied by high expression of mesenchymal genes including vimentin, and genes involved in the induction of epithelial‐mesenchymal transition (EMT; Neve *et al*., 2006; Sarrio *et al*., 2008).

Zinc finger E‐box binding protein 1 (ZEB1) and ZEB2 (also known as δEF‐1 and SIP‐1, respectively) are well‐known transcriptional regulators that induce EMT, which plays important roles in both normal physiological and pathological processes (Eger *et al*., 2005; Vandewalle *et al*., 2005; Zhang *et al*., 2015). While ZEB1 and ZEB2 have many similar properties in transcriptional regulation, they are different in their expression profiles, some molecular and biological functions, including regulation of cell differentiation and disease progression (Postigo and Dean, 2000; Wakamatsu *et al*., 2001). Epithelial cells lose their adhesive property and become migratory and invasive as they become mesenchymal cells during the EMT process (Nieto *et al*., 2016; Thiery *et al*., 2009). Transforming growth factor‐β (TGF‐β) is one of the main cytokines that promotes the EMT (Heldin *et al*., 2012; Lamouille *et al*., 2014). Specifically, TGF‐β binds to type I and type II receptors and transduces signals through Smad and non‐Smad signaling pathways (Derynck and Zhang, 2003; Heldin *et al*., 1997; Massague, 2012). The TGF‐β type I receptor is activated by ligand stimulation and induces the phosphorylation of the receptor‐regulated Smads (R‐Smads), Smad2 and Smad3, which form trimeric complexes with the common‐partner Smad, Smad4. These Smad complexes translocate into the nucleus where they regulate the transcription of various target genes in cooperation with other transcription factors. While expression of ZEB1 and ZEB2 is suppressed by epithelial miR‐200 family of miRNA (Gregory *et al*., 2008), TGF‐β induces their expression in addition to some other EMT‐related transcription factors, including Snail, and Slug, in certain types of normal and cancer cells (Gregory *et al*., 2011; Heldin *et al*., 2012; Miyazono *et al*., 2012; Xu *et al*., 2009).

We reported previously that TGF‐β decreases the expression of E‐cadherin through the induction of ZEB1 and ZEB2 in mouse mammary epithelial cells (Shirakihara *et al*., 2007). We also reported that it induces isoform switching of fibroblast growth factor receptors by alternative splicing, which occurs through downregulation of the expression of epithelial splicing regulatory proteins (ESRPs) by ZEB1 and ZEB2 (Horiguchi *et al*., 2012). Furthermore, the expression profiles of ZEB1 and ZEB2 are inversely correlated with those of ESRPs in human breast cancer cell lines and tumor specimens (Horiguchi *et al*., 2012). Consistent with the relationships between the EMT and cancer malignancy, high expression of ZEB1 is associated with poor prognosis of many types of cancer, including breast cancer (Chu *et al*., 2013; Jang *et al*., 2015). ZEB2 expression is also reported to be associated with poor prognosis of several types of cancer, although less frequently than ZEB1 (Fang *et al*., 2013; Prislei *et al*., 2015). It was recently reported that the EMT is involved in cancer malignancy by contributing not only to metastasis but also to the acquisition of cancer stem cell properties and chemoresistance (Fischer *et al*., 2015; Ye and Weinberg, 2015; Ye *et al*., 2015; Zheng *et al*., 2015). A recent genome‐wide analysis of EMT‐related transcription factor binding regions in pancreatic cancer cells suggested that ZEB1 plays a role in inducing the mesenchymal phenotype by suppressing enhancers that regulate the expression of epithelial genes (Diaferia *et al*., 2016). However, that analysis focused only on epithelial gene expression that was related to the EMT. Thus, the detailed mechanisms by which ZEB1 and ZEB2 contribute to poor prognosis in cancer remain to be elucidated.

Here, we employed chromatin immunoprecipitation sequencing (ChIP‐seq) and RNA sequencing (RNA‐seq) to investigate the transcriptional program that is regulated by ZEB1 in several basal‐type breast cancer cell lines. We found that ZEB1 directly upregulated the production of inflammatory cytokines in the basal‐type breast cancer cell lines MDA‐231‐D (a highly metastatic clone of MDA‐MB‐231; Ehata *et al*., 2007) and Hs578T. ZEB2 partially showed similar function, including the induction of interleukin (IL)‐6 and IL‐8 production. Our findings suggested that ZEB1 promotes the proliferation of cancer cells and contributes to the formation of the tumor microenvironment by regulating the expression of inflammatory cytokines. The ZEB1‐regulated inflammatory phenotype identified in this study provides insights into a mechanism that is critical for cancer progression and helps explain the poor prognosis of basal‐type breast cancer.

## 2 Materials and methods

### 2.1 Cell culture

The MDA‐231‐D human basal‐type breast cancer cell line, a highly bone metastatic clone of MDA‐MB‐231, was described previously (Ehata *et al*., 2007) and cultured in Dulbecco's modified Eagle's medium (DMEM; #11965, Thermo Fisher Scientific, Waltham, MA, USA). Hs578T human basal‐type breast cancer cells were obtained for use in this study from the American Type Culture Collection (ATCC, Manassas, VA, USA) and cultured in RPMI1640 (#11875, Thermo Fisher Scientific) with 0.01 mg·mL^−1^ of insulin (12585‐014, Thermo Fisher Scientific). HCC1954‐Luc human basal‐type breast cancer cells were obtained for use in this study from the Japanese Collection of Research Bioresources (JCRB) Cell Bank (Ibaragi, Osaka, Japan) and cultured in RPMI1640. MCF7 human luminal‐type breast cancer cells were obtained from JCRB and cultured in DMEM with 0.01 mg·mL^−1^ of insulin. 4T1 mouse breast cancer cells were obtained from the ATCC and cultured in high‐glucose DMEM. WI‐38 and IMR‐90 human lung fibroblast cells were obtained from the ATCC for use in this study and cultured in high‐glucose DMEM. Hs578T, HCC1954‐Luc, MCF7, WI‐38, and IMR‐90 cells were used within 6 months of passage after purchase. All culture media included 10% fetal bovine serum (FBS), 100 units·mL^−1^ penicillin G, and 100 μg·mL^−1^ streptomycin. All cells were maintained in a 5% CO_2_ atmosphere at 37 °C.

### 2.2 Antibodies and reagents

The following antibodies were used for immunoblotting: anti‐pSTAT3 Tyr705 (D3A7, #9145; Cell Signaling Technology, Danvers, MA, USA), anti‐STAT3 (124H6, #9139; Cell Signaling Technology), anti‐FLAG M2 (F3165; Sigma‐Aldrich, St. Louis, MO, USA), anti‐α‐tubulin (DM1A, T6199; Sigma‐Aldrich), anti‐β‐actin (AC‐15, A5441; Sigma‐Aldrich), anti‐ZEB1 (NBP1‐05987; Novus Biologicals, Minneapolis, MN, USA), and anti‐ZEB2 antibodies (A302‐474A; BETHYL Laboratories, Montgomery, TX, USA). The anti‐ZEB1 antibody (NBP1‐05987) was also used for chromatin immunoprecipitation. Recombinant human TGF‐β (TGF‐β3) and human IL‐6 were obtained from R&D systems (Minneapolis, MN, USA). LY364947 was from Calbiochem, Merck Millipore (Billerica, MA, USA). Cells were not serum‐starved during ligand stimulation.

### 2.3 RNA interference

Transfection of Stealth Select siRNA (Thermo Fisher Scientific) was performed according to the recommended protocol using Lipofectamine RNAiMAX (Thermo Fisher Scientific). We used two sets of ZEB1, ZEB2, and IL‐6 siRNA: ZEB1 (HSS110548 and HSS186235), ZEB2 (HSS114854 and HSS190654), and IL‐6 (HSS105338 and HSS105339). Control siRNA (Medium GC Complex #2: 12935‐112) was purchased from Thermo Fisher Scientific.

### 2.4 RNA‐seq and data analysis

RNA‐seq was performed as described previously (Isogaya *et al*., 2014; Mizutani *et al*., 2016). cDNA libraries were prepared using the RNeasy Mini Kit with the On‐Column DNase Digestion Set (QIAGEN, Venlo, The Netherlands), Dynabeads mRNA DIRECT Purification Kit, and the Ion Total RNA‐Seq Kit v2 (Thermo Fisher Scientific).

### 2.5 Chromatin immunoprecipitation, ChIP‐seq, and data analysis

MDA‐231‐D, Hs578T, and MCF7 cells were cultured in 10‐cm plates, and ChIP and ChIP‐seq were performed as described previously (Koinuma *et al*., 2009; Murai *et al*., 2015). Data were obtained using the Ion Proton sequencer (Thermo Fisher Scientific). Unfiltered 50‐bp sequence reads were aligned against the human reference genome (NCBI Build 36, hg19). Public anti‐ZEB1 ChIP‐seq data were obtained from GEO (GSM1574278, GSM1010809, GSM803411; ENCODE Project Consortium, 2012). Peaks were called using MACS2 (Zhang *et al*., 2008). cisgenome software was used to assign a binding site to the nearest gene within 50 kb of a peak (Ji *et al*., 2011).

### 2.6 RNA isolation and quantitative RT‐PCR (qRT‐PCR)

Total RNA was extracted using the RNeasy Mini Kit (QIAGEN). First‐strand cDNA synthesis was performed using PrimeScript2 reverse transcriptase and oligo dT primers (TaKaRaBio, Shiga, Japan) according to the manufacturer's instructions. qRT‐PCR was performed using the ABI PRISM7500 Fast Real‐Time PCR System or the StepOnePlus Real‐Time PCR system (Thermo Fisher Scientific) and the Fast Start Universal SYBR Green Master Mix with ROX (Roche Diagnostics, Basel, Switzerland). Mouse and human *GAPDH* were used for normalization. The primer sequences are shown in Table S1. Data are reported as the means of two technical replicates unless otherwise indicated in the figure legends.

### 2.7 Preparation of conditioned medium and enzyme‐linked immunosorbent assay (ELISA)

MDA‐231‐D cells and Hs578T cells were seeded (2 × 10^5^ per well in six‐well plates for IL‐6 experiments and 1 × 10^5^ per well in 12‐well plates for IL‐8 experiments). After overnight incubation, siRNA was transfected as described previously, followed by TGF‐β incubation (1 ng·mL^−1^), LY364947 treatment (1 μm), or a medium change (2 mL per well for six‐well plates and 1 mL per well for 12‐well plates) on the next day of transfection. The supernatant was collected after incubation for 48 h. To prepare the supernatant from HCC1954‐Luc cells, the cells were seeded on a six‐well plate (1 × 10^5^ per well), followed by TGF‐β stimulation, LY364947 (3 μm) treatment, or a medium change (2 mL) the next day. After 48 h of incubation, the supernatant was collected. The concentrations of IL‐6 and IL‐8 were measured using the human IL‐6 Quantikine ELISA Kit and the human CXCL8/IL‐8 Quantikine ELISA Kit (R&D systems), respectively, according to the manufacturer's instructions. Data are reported as the means of two biological replicates.

### 2.8 Lentiviral vector preparation and infection, and construction of plasmids

Lentiviral expression vectors were obtained from Hiroyuki Miyoshi (RIKEN BioResource Center; present address: Keio University, Tokyo, Japan). Lentiviral vectors were prepared by cotransfection of 293FT cells with pCSII‐EF‐mZEB1 or pCS‐CDF‐CG‐PRE (for EGFP expression) and packaging vectors (pCAG‐HIVgp and pCMV‐VSV‐G‐RSV‐Rev). The medium was changed after 24 h of transfection, and the culture media containing virus particles were collected after incubation for an additional 48 h. cDNAs encoding mouse ZEB1 and human ZEB2 were cloned into lentiviral expression vector or pcDEF3 expression vector. These plasmids were introduced into cells using Lipofectamine 2000 or Lipofectamine 3000 (Thermo Fisher Scientific) according to the recommended protocols.

### 2.9 Antibody array

The Human Cytokine Antibody Array C2000 (Ray Biotech, Norcross, GA, USA) was used according to the manufacturer's instructions. The LAS‐4000 lumino‐image analyzer (GE Healthcare, Buckinghamshire, UK) was used for chemiluminescence detection, and the strength of each spot was measured using the line profile function of MultiGauge software (FUJIFILM, Tokyo, Japan) and analyzed using the Analysis Tool for AAH‐CYT‐2000 (Ray Biotech).

### 2.10 Immunoblotting

RIPA buffer (50 mm Tris/HCl (pH 8.0), 150 mm NaCl, 1% NP‐40, 0.1% SDS, and 0.5% sodium deoxycholate) or NP‐40 lysis buffer (1% NP‐40, 150 mm NaCl, 20 mm Tris/HCl pH 7.5) that included Complete EDTA‐free protease inhibitor cocktail (Roche Diagnostics) and Phosphatase Inhibitor Cocktail (EDTA‐free; Nacalai Tesque, Kyoto, Japan) was used for cell lysis. The same amount of proteins was applied to the gels for protein analysis. SDS gel electrophoresis and immunoblotting were performed as described previously (Koinuma *et al*., 2011), and the signals were analyzed using the LAS‐4000 lumino‐image analyzer. Membranes were incubated with the primary antibodies at 4 °C overnight.

### 2.11 Tissue array

A tissue array of multiple organ tumors (MC6163) was purchased from US Biomax (Rockville, MD, USA). The paraffin‐embedded array was deparaffinized and rehydrated followed by antigen retrieval using 10 mm sodium citrate buffer (pH 6.0). Endogenous peroxidase activity was blocked by 3.0% hydrogen peroxide. The array was then blocked with Blocking One reagent (Nacalai Tesque) and incubated with anti‐ZEB1 antibody (NBP1‐05987; Novus Biologicals) and human IL‐6 antibody (R&D systems). Anti‐rabbit Alexa488 and anti‐goat Alexa594 were used as the secondary antibodies. The array was mounted with DAPI‐containing mounting medium. Tile scanning was performed using the Leica DMI6000 B inverted microscope with adaptive focus control (Leica Microsystems, Wetzlar, Germany). The signal intensity of each array spot was scored by two researchers (A.K. and Y.T.).

### 2.12 Tumor model

All animal experiments were performed in accordance with the policies of the animal ethics committee of the University of Tokyo. HCC1954‐Luc cells (4 × 10^5^) were injected into the mammary fat pads of 6‐week‐old female BALB/c nude mice, and 4T1 cells (5 × 10^5^) were injected subcutaneously into BALB/c mice. The lengths and widths of the resulting tumors were measured using calipers, and the tumor volume was calculated as follows: 0.5 × (major axis) × (minor axis)^2^.

### 2.13 Senescence‐associated β‐galactosidase (SA‐βGal) staining

Senescent cells were detected using the Senescence β‐Galactosidase Staining Kit (Cell Signaling Technology) according to the manufacturer's instructions.

### 2.14 Flow cytometry

Primary tumors were isolated from mice, cut into pieces, and digested in RPMI1640 containing 200 U·mL^−1^ of collagenase, type I (Worthington, Lakewood, NJ, USA) and 10 μg·mL^−1^ of DNaseI (Roche Diagnostics) for 60 min at 37 °C on a shaking platform. The samples were then washed and filtered through a cell strainer (100‐μm nylon; Corning, Corning, NY, USA). Red blood cells were lysed in Red Blood Cell Lysis Buffer (Roche Diagnostics). The collected cells were incubated with FcR blocking reagent, mouse (Miltenyi Biotec, Bergisch Gladbach, Germany), for 15 min on ice. For flow cytometry, the cells were stained with the following antibodies for 30 min on ice in the dark: CD11b‐APC (M1/70) and Ly6C‐PE (HK1.4) from eBioscience (San Diego, CA, USA), Ly6G‐PE/Cy7 (1A8) and CD45‐Alexa Fluor 700 (30‐F11) from BioLegend (San Diego, CA, USA), and Gr‐1‐FITC (RB6‐8C5) from BD Biosciences (San Jose, CA, USA). The cells were analyzed by Gallios Flow Cytometer (Beckman Coulter, Fullerton, CA, USA) and were further analyzed using flowjo software (TreeStar software, Ashland, OR, USA).

### 2.15 Isolation and stimulation of mouse bone marrow cells

The femurs and tibias of 6‐ to 8‐week‐old C57BL/6J mice were isolated, and the marrow was flushed with HBSS using a 27‐gauge needle (Thermo Fisher Scientific). Then, the cells were filtered through a cell strainer (100‐μm nylon), and the red blood cells were lysed. Cells (2.5 × 10^6^) were incubated with RPMI1640 supplemented with 2 mm l‐glutamine (Thermo Fisher Scientific), 10 mm HEPES (Thermo Fisher Scientific), 20 μm 2‐mercaptoethanol (Thermo Fisher Scientific), 100 units·mL^−1^ penicillin G, 100 μg·mL^−1^ streptomycin, and nonessential amino acids (NEAA; Sigma‐Aldrich) plus 50% HCC‐GFP‐ or HCC‐ZEB1‐conditioned media. After 20 min, the cells were lysed and subjected to immunoblot analysis.

### 2.16 Statistical analysis

Student's *t*‐test was used for two‐sample analyses, and the Tukey–Kramer test was used for multisample analyses. Mann–Whitney *U*‐test was used for *in vivo* data.

## 3 Results

### 3.1 Identification of ZEB1 target genes in breast cancer cells

**Figure 1:**
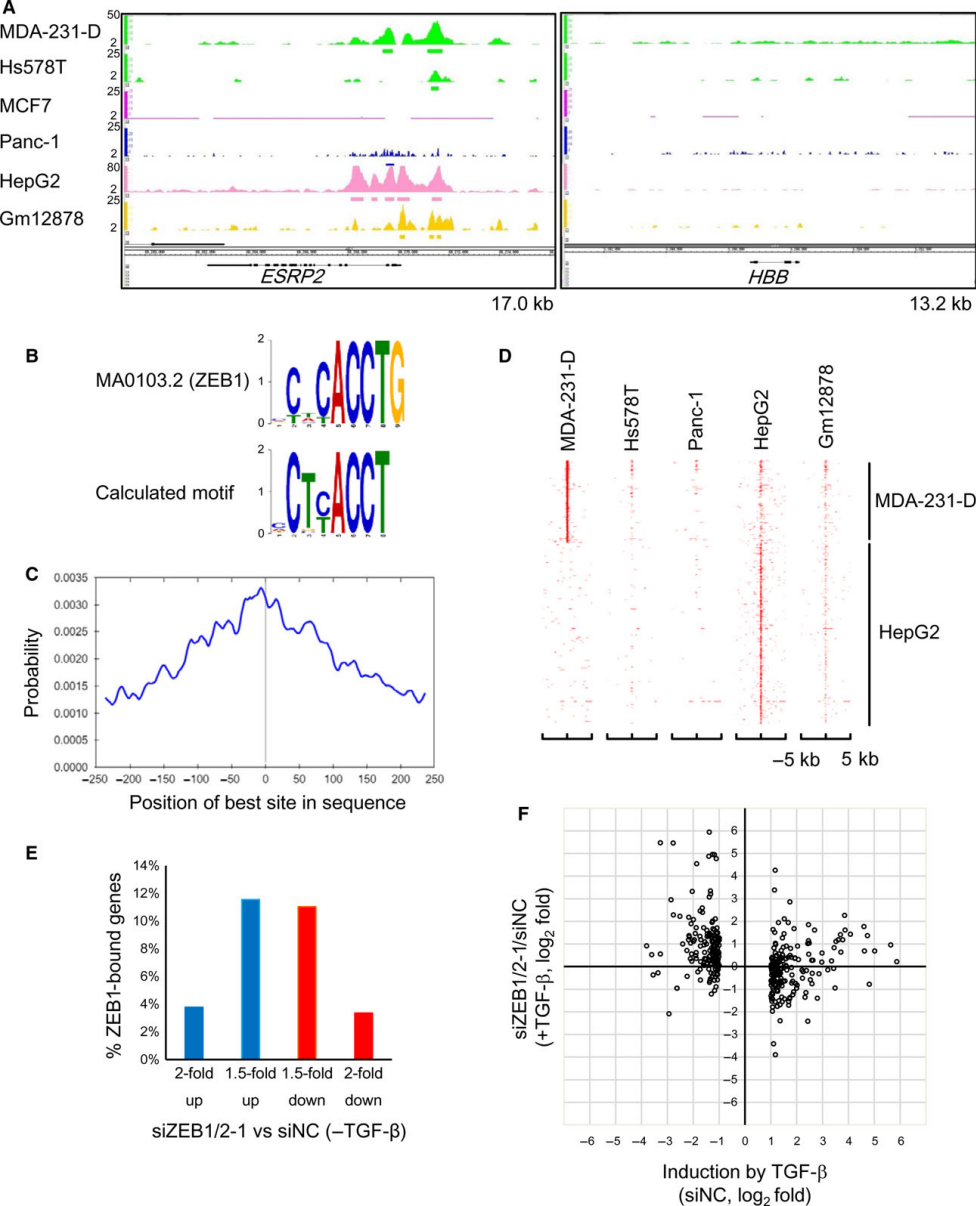
Identification of ZEB1‐binding DNA regions. (A) ZEB1‐binding signals at the *ESRP2* and *HBB* gene loci. The *y*‐axis scales are not equal as they were adjusted to show the binding signals clearly for each cell line. The kb sizes denote the ranges shown in the panels. The bars below the binding signals indicate the significant binding regions (*q* < 0.05). (B) *De novo* motif prediction was performed from the 500‐bp sequences flanking the summit position of each ZEB1‐binding region using the Gibbs Motif Sampler provided by CisGenome. Default parameters were used except for the mean motif length (8), maximum motif length (15), and K (15). The identified E‐box‐like motif was compared to known motifs using the Tomtom motif comparison tool in MEME suite version 4.11.2. The alignment of the calculated motif with a top‐ranked motif (MA0103.2) that was deposited as a ZEB1‐binding site is shown. *Y*‐axis scale: bits. (C) Motif centrality analysis was performed using the motif calculated in (B) and CentriMo of the MEME suite. The *x*‐axis indicates the relative position of the best site from the peak summit of each ZEB1‐binding region. (D) A heatmap representation of the ZEB1‐binding regions in the genomes of the indicated cell lines. The *y*‐axis indicates the union of the ZEB1‐binding regions that were obtained from five cell lines as sorted by the signal intensities of MDA‐231‐D and HepG2. *X*‐axis: the position of each binding region relative to the peak summits of the ZEB1‐binding regions shown on the *y*‐axis. (E) Frequencies of the ZEB1‐bound genes showing expression that was up‐ or downregulated more than 1.5‐ or 2‐fold by siRNA targeting ZEB1 and ZEB2 (siZEB1‐1 and siZEB2‐1) in the absence of TGF‐β. Of the 15 175 ZEB1‐bound genes (ZEB1‐binding regions within 50 kb from the transcription start site, TSS), the genes encoding small RNA or showing expression that was < 10 FPKM (fragments per kilobase of exon per million mapped reads) were excluded from the evaluation. In panels (E) and (F), MDA‐231‐D cells were transfected with siRNA and treated with or without 1 ng·mL^−1^ of TGF‐β for 24 h. RNA‐seq was then performed. siNC, control siRNA. (F) A scatter plot showing the relationship between the effect of ZEB1/2 siRNA (siZEB1‐1 and siZEB2‐1) and TGF‐β as determined by RNA‐seq. ZEB1‐bound, TGF‐β‐regulated genes showing expression that was induced or suppressed ≥ 2‐fold were selected (*n* = 5975). siZEB1/2‐1, siZEB1‐1 + siZEB2‐1. Each dot represents a single gene.

To determine the genome‐wide distribution of ZEB1‐binding regions in MDA‐231‐D and Hs578T basal‐type breast cancer cells, we performed ChIP‐seq analysis using a validated ZEB1 antibody that did not cross‐react with ZEB2 (Fig. S1A; Horiguchi *et al*., 2012). We also obtained ChIP‐seq data in MCF7 luminal‐type breast cancer cells. Data were obtained from TGF‐β‐treated cells based on the known functional interaction between Smad signaling and ZEB1 and ZEB2 (Postigo, 2003; Postigo *et al*., 2003; Verschueren *et al*., 1999). We identified 32 907 binding regions in MDA‐231‐D cells, 13 514 regions in Hs578T cells, and 281 regions in MCF7 cells that had *q*‐values < 0.05. Using a more stringent threshold for peak calling, 14 811, 3131, and 108 binding regions were identified in MDA‐231‐D, Hs578T, and MCF7 cells, respectively (*q* < 10^−4^). Significant peaks were found at known binding sites (*ESRP2* and *CDH1* gene loci) in the basal‐type breast cancer cells (Horiguchi *et al*., 2012), but not at the *HBB* gene locus, which served as a negative control (Fig. 1A and data not shown). No peaks were found at the *ESRP2* and *CDH1* gene loci in MCF7 cells, which likely reflected the low expression of ZEB1 in luminal‐type breast cancer cells (Horiguchi *et al*., 2012). One of the *de novo* predicted common motifs in the ZEB1‐binding regions in MDA‐231‐D cells matched the known ZEB1‐binding motif, which contained ‘CACCT’ (*q* = 0.0289) and was found in 38% of the 14 811 binding regions (Fig. 1B). The identified motif was also enriched toward the peak summit positions of ZEB1‐binding regions, supporting the validity of the obtained ChIP‐seq data (Fig. 1C).

Our data were then compared to all of the publically available human ZEB1‐binding data for human pancreatic carcinoma Panc‐1, human hepatoblastoma HepG2, and the Gm12878 B‐lymphoblastic cell lines using the same read aligner, peak caller, and parameters (Diaferia *et al*., 2016; Hensen *et al*., 2014). We found that MDA‐231‐D cells and HepG2 cells had distinct profiles in terms of their ZEB1‐binding regions and that Hs578T and Panc‐1 cells, but not Gm12878 cells, shared ZEB1‐binding regions with MDA‐231‐D cells (Fig. 1D). Many of the ZEB1‐binding regions in Hs578T and Panc‐1 cells overlapped with those in MDA‐231‐D cells (69.8% and 71.7%, respectively). In contrast, Gm12878 cells shared only 29.3% of their ZEB1‐binding regions with MDA‐231‐D cells, and most of the remaining regions were shared with HepG2 cells, indicating that ZEB1‐binding regions were relatively conserved in basal‐type breast cancer cells, but not in other types of cancer cells.

ZEB1 acts as both a transcriptional activator and repressor (Lehmann *et al*., 2016; Sanchez‐Tillo *et al*., 2011). To clarify the global view of ZEB1‐regulated transcription, we performed RNA‐seq using MDA‐231‐D cells. To examine the function of ZEB1 in the context of the EMT, MDA‐231‐D cells were transfected with a combination of siRNA targeting ZEB1 and ZEB2 and were then left untreated or were stimulated with TGF‐β. The amounts of ZEB1 and ZEB2 proteins were efficiently decreased by the siRNA in MDA‐231‐D cells (Fig. S1B) and the specificity of the siRNA was confirmed (Fig. S1C). The ZEB1‐binding regions were present within 50 kb of the transcriptional start site of 14 900 genes, which accounted for 64.0% of the total number of genes analyzed. Of the ZEB1‐bound genes, 5975 were expressed at 10 or more FPKM (fragments per kilobase of exon per million mapped reads), and the number of genes that were either upregulated or downregulated by ZEB1/2 knockdown was similar (Fig. 1E).

We then evaluated the effect of ZEB1/2 siRNA on TGF‐β‐induced changes in the expression of ZEB1‐bound genes. Focusing on the ZEB1‐target genes that were either up‐ or downregulated more than 2‐fold by TGF‐β, we found that knockdown of ZEB1/2 largely upregulated the expression of genes that were downregulated by TGF‐β (Fig. 1F). This tendency was not observed for genes that were upregulated by TGF‐β.

### 3.2 ZEB1 regulates the expression of inflammatory response genes

**Figure 2:**
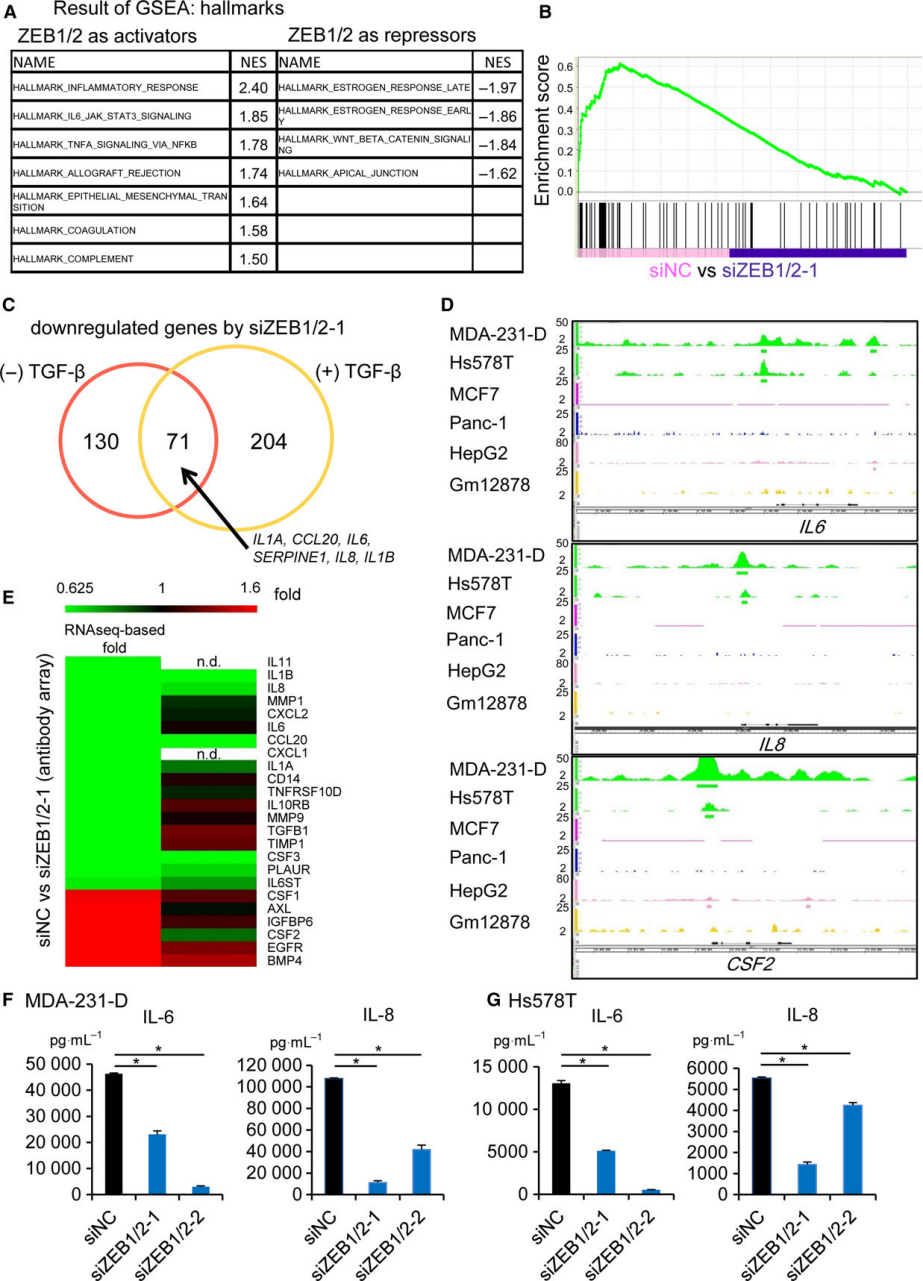
ZEB1 regulates the expression of inflammatory response genes. (A–C) GSEA was performed using the RNA‐seq data in Fig. 1E and F. ZEB1‐bound genes were selected for evaluation. (A) Gene sets in MDA‐231‐D cells that were differentially expressed after treatment with ZEB1/2 siRNA (siZEB1‐1 and siZEB2‐1). The most enriched MSigDB hallmark signatures that had normalized enrichment scores (NES) > 1.5 or < −1.5 are listed. (B) Enrichment plot of the HALLMARK_INFLAMMATORY_RESPONSE gene set. ZEB1‐bound genes (vertical black bars in the bottom panel) were enriched toward the left; ZEB1 and ZEB2 functioned as transcriptional activators. siZEB1/2‐1, siZEB1‐1 + siZEB2‐1. (C) Venn diagram showing the overlap of genes that were downregulated by ZEB1/2‐1 siRNA in the presence and absence of TGF‐β. Genes with maximum FPKM values ≥ 10 in the four samples were selected for further evaluation, and those with fold changes (FPKM (siZEB1/2) + 0.01)/(FPKM(siNC) + 0.01) ≤ 0.5 were defined as downregulated genes. (D) Visualization of the ZEB1 ChIP‐seq data tracks at the *IL6*,*IL8*, and *CSF2* gene loci. Data are shown as in Fig. 1A. (E) Antibody array analysis of proteins in conditioned medium obtained from serum‐starved MDA‐231‐D cells. ZEB1‐bound genes and genes regulated by ZEB1/2 siRNA according to ChIP‐seq and RNA‐seq analysis are shown in a heat map in the order of downregulation by siRNA (left panel). Conditioned medium was collected from MDA‐231‐D cells treated with ZEB1/2‐1 siRNA and analyzed using an antibody array. The effects of ZEB1/2 siRNA on cytokine secretion are shown in the right panel. Genes were selected according to the following criteria: Maximal expression values (FPKM) were ≥ 10, and there was a fold change ≥ 1.5 in response to ZEB1/2 siRNA. Note that the amount of IL‐6 protein in this cell line was very high and could not be quantitatively evaluated in this array. n.d.: not detected. (F) The effect of ZEB1/2 siRNA on the secretion of IL‐6 and IL‐8 by MDA‐231‐D cells as determined by ELISA. Error bars indicate the S.D. *P < 0.05. (G) The effect of ZEB1/2 siRNA on the secretion of IL‐6 and IL‐8 by Hs578T cells as determined by ELISA. Error bars indicate the S.D. *P < 0.05. In (F) and (G), siZEB1/2‐1, siZEB1‐1 + siZEB2‐1; siZEB1/2‐2, siZEB1‐2 + siZEB2‐2.

To identify the functional signatures of ZEB1‐bound genes that were enriched in MDA‐231‐D cells, gene set enrichment analysis (GSEA) was conducted using MDA‐231‐D cell gene expression data (Fig. 2A). The most notable finding was that the expression of inflammatory response genes was downregulated by the knockdown of ZEB1 and ZEB2 in the TGF‐β‐untreated condition (Fig. 2B). The downregulated genes that were categorized as inflammatory response genes are listed in Table S2, along with positional information about ZEB1 binding. A similar tendency was observed in the TGF‐β‐treated condition; remarkably, the *IL1A*,* CCL20*,* IL6*,* SERPINE1, IL8*, and *IL1B* genes were commonly and strongly downregulated in both conditions (Fig. 2C). Focusing on the ZEB1 ChIP‐seq data, peaks were found at the promoter or enhancer regions of the identified genes, including *IL6*,* IL8*, and *CSF2*, in MDA‐231‐D and Hs578T cells, but not in MCF7 cells (Fig. 2D), suggesting that ZEB1 binds and regulates the expression of inflammatory response genes in basal‐type breast cancer cells.

Based on these findings, we focused on ZEB1‐regulated secretory proteins. An antibody array detected 174 human cytokines in the conditioned culture media of MDA‐231‐D cells that were transfected with ZEB1/2 siRNA. ZEB1/2 siRNA downregulated secreted IL‐1β, IL‐8, and IL‐1α proteins in MDA‐231‐D cells. The expression levels of the CCL20, PLAUR (plasminogen activator, urokinase receptor), CSF3 (granulocyte colony‐stimulating factor, G‐CSF), and CSF2 (granulocyte–macrophage colony‐stimulating factor, GM‐CSF) proteins were also regulated by ZEB1 (Fig. 2E). Of note, reduction in secreted IL‐6 protein by ZEB1/2 siRNA was not observed because of the saturated signals in both control siRNA‐ and ZEB1/2 siRNA‐treated conditions. We then used two sets of ZEB1/2 siRNA to quantitatively evaluate the effects of ZEB1/2 siRNA on IL‐6 and IL‐8 secretion by MDA‐231‐D cells and Hs578T cells using ELISA and found a similar tendency as in the RNA‐seq analysis (Fig. 2F and G). Notably, the amounts of proteins secreted by MDA‐231‐D cells were comparable to the levels secreted by inflammatory cells, suggesting that IL‐6 and IL‐8 were produced at functional levels (Nastasi *et al*., 2015; Pazmandi *et al*., 2012).

### 3.3 ZEB1 and ZEB2 have similar functions in the regulation of inflammatory cytokine expression

**Figure 3:**
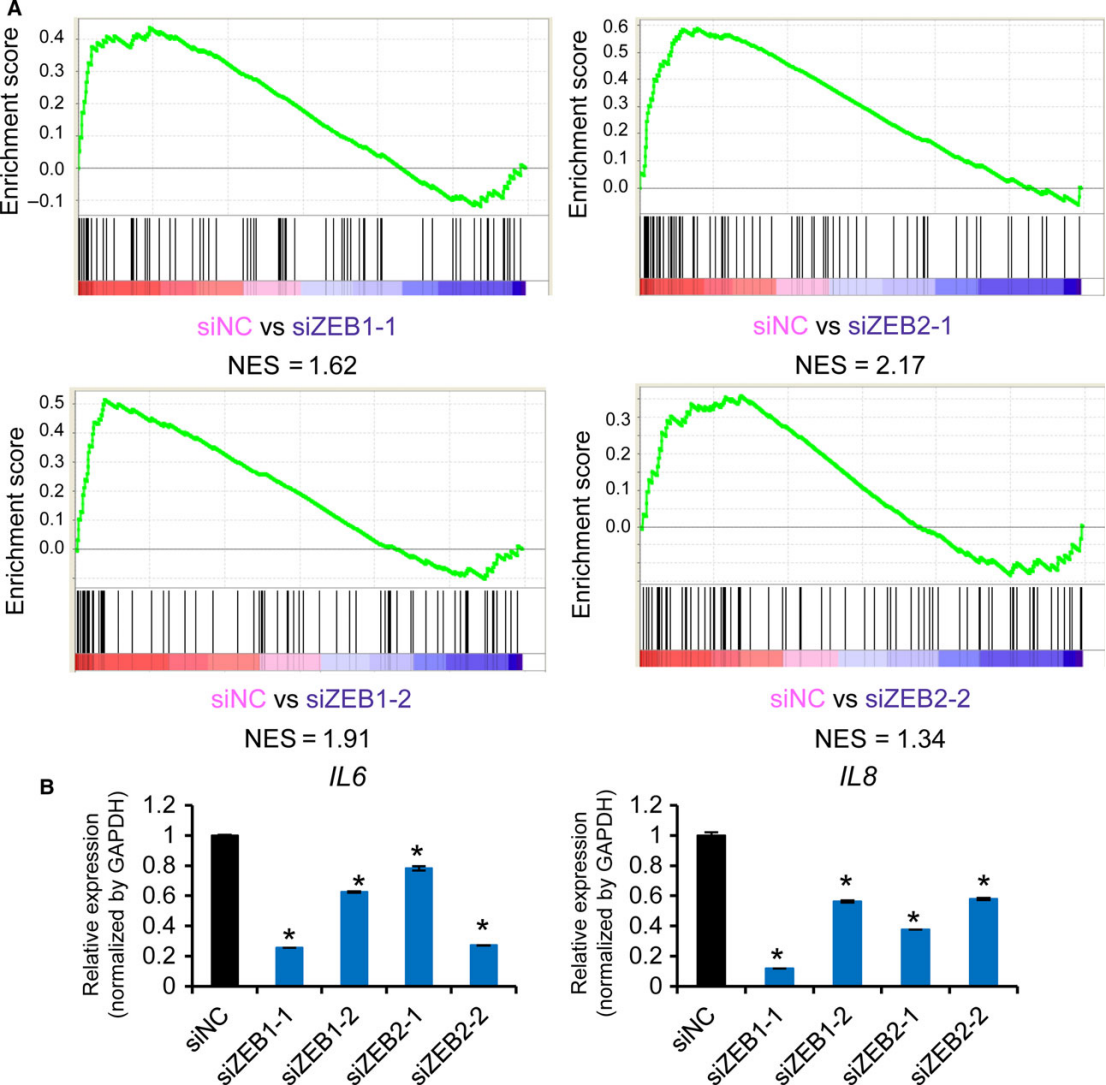
ZEB1 and ZEB2 have similar functions in the regulation of inflammatory cytokines. (A) Enrichment plots of the HALLMARK_INFLAMMATORY_RESPONSE gene set showing the effect of ZEB1 or ZEB2 siRNA in MDA‐231‐D cells. List of genes (vertical black bars in the bottom panel) were the same as Fig. 2B and enriched toward the left; ZEB1 and ZEB2 functioned as transcriptional activators. (B) Effect of ZEB1 or ZEB2 siRNA on the expression of *IL6* and *IL8* in MDA‐231‐D cells determined by qRT‐PCR. Error bars, S.D. *P < 0.05 compared to siNC.

While both ZEB1 and ZEB2 suppress the expression of *CDH1* and induce EMT, previous reports have also revealed some different functions between them. We thus evaluated whether ZEB1 and ZEB2 have similar functions in the production of inflammatory cytokines. We knocked down the expression of ZEB1 or ZEB2 and obtained RNA‐seq data from MDA‐231‐D cells. Knockdown of ZEB1 or ZEB2 did not strongly affect each other's protein expression (Fig. S1C). GSEA analysis suggested that siRNA against either ZEB1 or ZEB2 downregulated the expression of inflammatory response genes, including *IL6* and *IL8*, although normalized enrichment score (NES) of inflammatory response genes in one of the ZEB2 siRNA was not more than 1.5 (NES = 1.34; Fig. 3A and Fig. S1D). qRT‐PCR analysis also revealed that expression of *IL6* and *IL8* was decreased by knockdown of either ZEB1 or ZEB2 (Fig. 3B). Therefore, ZEB1 and ZEB2 have essentially similar functions in the regulation of inflammatory response gene expression, especially *IL6* and *IL8*, in the basal‐type breast cancer cells.

### 3.4 TGF‐β affects the production of cytokines and chemokines that are regulated by ZEB1

**Figure 4:**
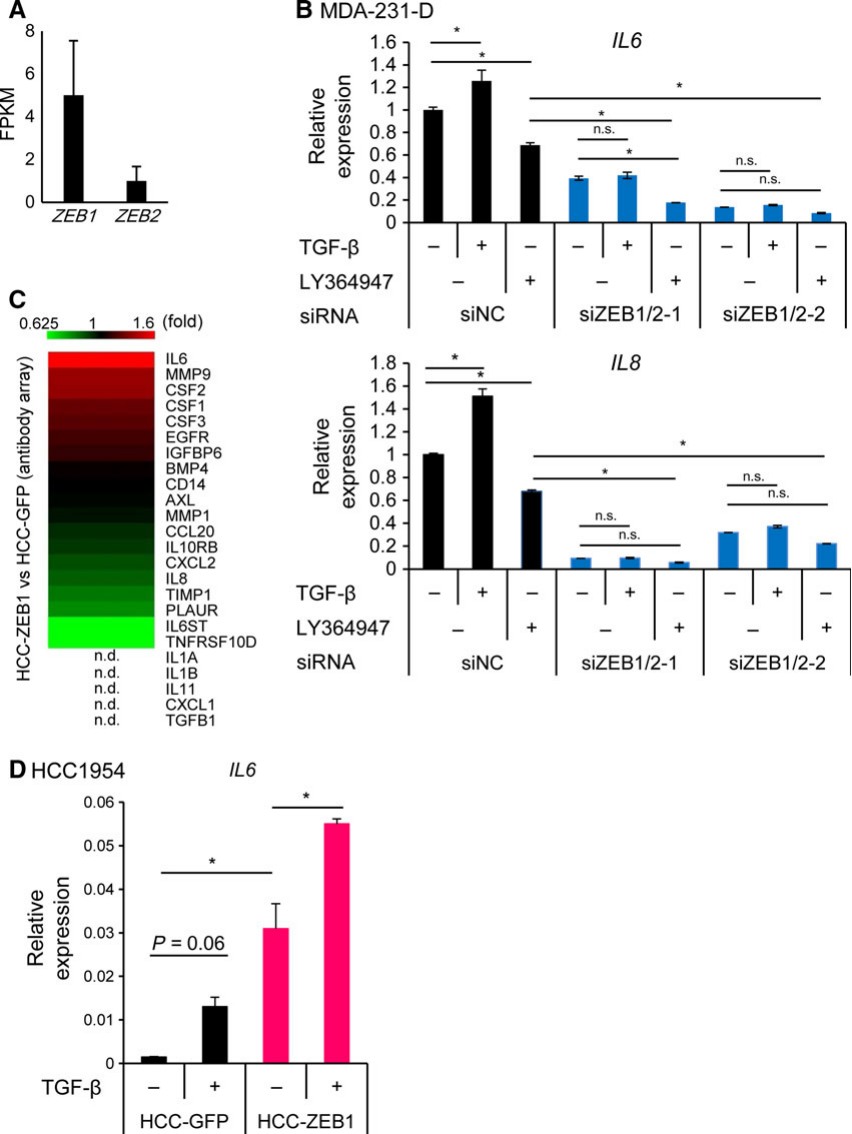
The effect of TGF‐β signaling on the expression of ZEB1‐regulated secretory proteins. (A) The expression of *ZEB1* and *ZEB2* in MDA‐231‐D cells as determined by RNA‐seq. Error bars, C.I. (B) qRT‐PCR analysis of *IL6* and *IL8* expression in the presence or absence of TGF‐β (1 ng·mL^−1^, 48 h) and a TGF‐β inhibitor (LY364947: 1 μm, 48 h) in MDA‐231‐D cells transfected with ZEB1/2 siRNA. Data represent the means of two biological replicates. Error bars, S.D. *P < 0.05; n.s., not significant. siZEB1/2‐1, siZEB1‐1 + siZEB2‐1; siZEB1/2‐2, siZEB1‐2 + siZEB2‐2. (C) A heatmap showing the effect of exogenous ZEB1 expression versus control vector infection as determined by antibody array analysis of the conditioned medium obtained from a 48‐h culture of serum‐starved HCC1954‐Luc cells. The list of proteins is the same as in Fig. 2E, and it is arranged in the order of induction by ZEB1 in HCC1954‐Luc cells. HCC‐ZEB1, ZEB1‐expressing HCC1954‐Luc cells; HCC‐GFP, GFP‐expressing HCC1954‐Luc cells. n.d., not detected using the same criteria as in Fig. 2E. (D) qRT‐PCR analysis to determine the effects of TGF‐β on *IL6* expression in HCC1954‐Luc cells that were overexpressing ZEB1. RNA was obtained from the cells 48 h after TGF‐β stimulation. Data are shown as the means of two biological replicates. Error bars, S.D. *P < 0.05.

ZEB1 functions as a transcriptional activator by binding to Smad1, Smad2, and Smad3 and supports their transcriptional activities (Postigo, 2003; Postigo *et al*., 2003), while ZEB2 inhibits TGF‐β signaling (Verschueren *et al*., 1999). Based on the similar functions of ZEB1 and ZEB2 on the expression of inflammatory cytokines, we then investigated whether ZEB1 and ZEB2 regulate the expression of inflammatory response genes induced by TGF‐β stimulation. Of the basal‐type breast cancer cell lines, MDA‐231‐D and Hs578T expressed ZEB1 and ZEB2, while HCC1954‐Luc cells showed only small amounts of ZEB1 and ZEB2 proteins (Fig. S2A; Horiguchi *et al*., 2012). In agreement with the immunoblot data, quantitative evaluation using RNA‐seq data in MDA‐231‐D cells suggested that the expression of *ZEB2* was lower than that of *ZEB1* (Fig. 4A). Of note, *ZEB1* and *ZEB2* expression was not upregulated by TGF‐β in MDA‐231‐D cells (Fig. S2B).

We found that TGF‐β increased the expression of *IL6* and *IL8*, while LY364947, which is a TGF‐β type I receptor kinase inhibitor, decreased their baseline expression levels (Fig. 4B). The effect of TGF‐β on the expression of *IL6* and *IL8* was not observed in the absence of ZEB1 and ZEB2. A similar tendency was observed regarding the TGF‐β‐induced expression of *CSF2* and *CXCL5* (Fig. S2B). Significant induction of *IL1B* by TGF‐β could not be seen; however, the effect of LY364947 on *IL1B* expression in the control siRNA‐transfected cells was inhibited by ZEB1/2 siRNA. Consistent results were not obtained regarding the effect of ZEB1/2 siRNA on TGF‐β‐induced *CXCL1* expression.

We then exogenously expressed ZEB1 and ZEB2 in HCC1954‐Luc cells by transfection of expression plasmids (Fig. S2C), and found that ZEB1, and to a lesser extent ZEB2, increased the *IL6* and *IL8* expression. We also established HCC‐1954‐Luc cells stably expressing ZEB1 (HCC‐ZEB1; Fig. S2D). Analysis of secreted proteins from HCC‐ZEB1 cells using an antibody array revealed that ZEB1 induced the production of IL‐6 and CSF2, but not IL‐8, in this cell line (Fig. 4C). *IL6* expression in HCC1954‐Luc cells was significantly increased by exogenous ZEB1 expression, similar to its effect in MDA‐231‐D cells (Fig. 4D). In addition, TGF‐β increased the expression of *IL6* in the presence of ZEB1 in HCC‐1954‐Luc cells (Fig. 4D). These results suggested that ZEB1 and possibly ZEB2 played a central role in *IL6* and *IL8* transcription independent of TGF‐β signaling, and simultaneously, they were required for the induction of *IL6* and *IL8* by TGF‐β. These results also suggested that production of inflammatory cytokines by ZEB1 and ZEB2 is induced in a context‐dependent manner and that expression of ZEB1 and ZEB2 is not sufficient to induce some of the target proteins.

### 3.5 Correlation of the expression of IL‐6 and IL‐8 with ZEB1 expression in various types of cancer

**Figure 5:**
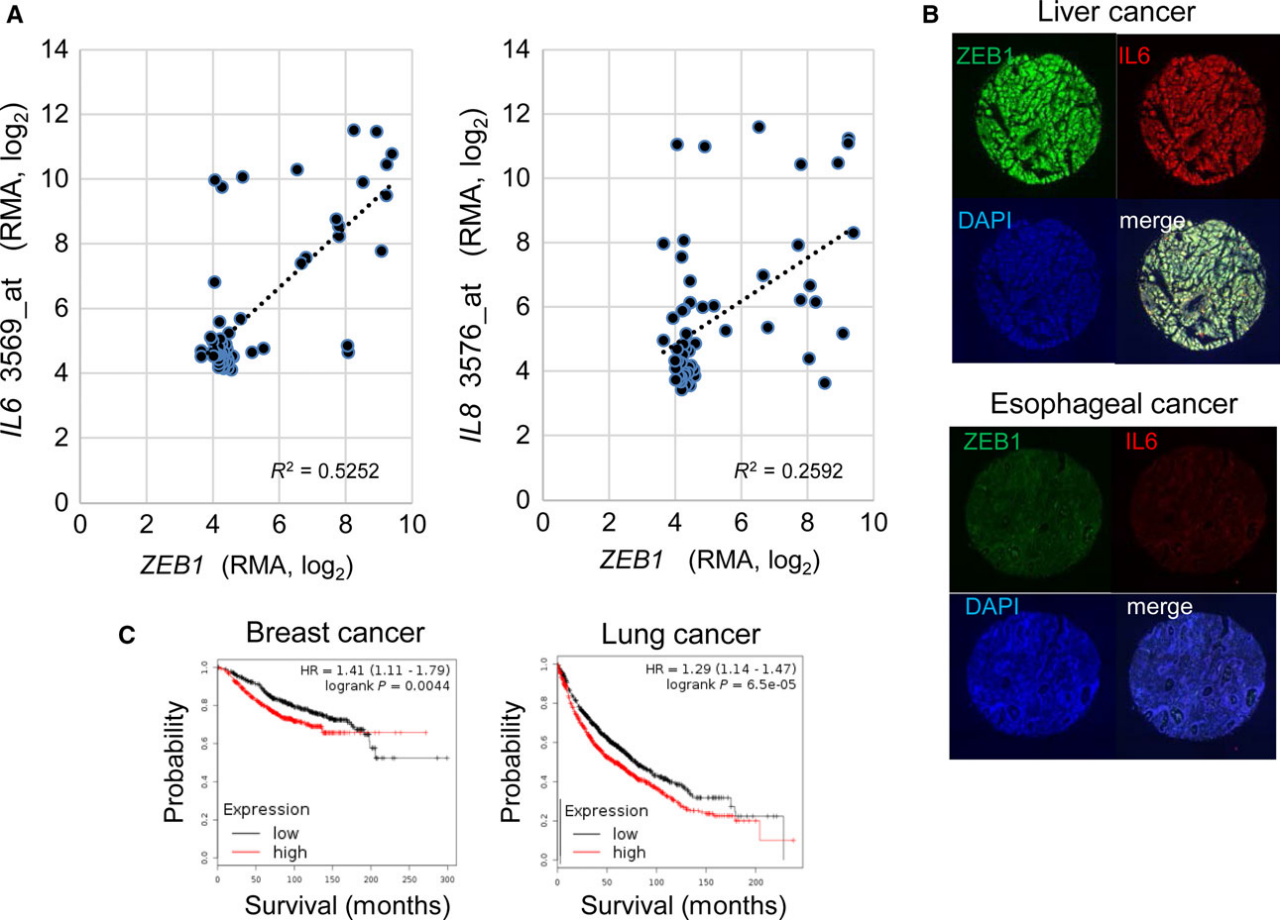
Correlation of the expression of ZEB1 and IL‐6 and other cytokines in various cancers. (A) Correlation analysis of the expression of *ZEB1* and cytokines in breast cancer cell lines using data obtained from the Cancer Cell Line Encyclopedia (CCLE). Breast cancer cell lines were selected for the analysis, and each dot represents a cell line. Affymetrix microarray probe IDs for *IL6* and *IL8* are shown after the gene symbols on the *y*‐axis. RMA, robust multiarray average. (B) Representative results obtained from the tissue array analysis in Fig. S3. The top panel shows a case that was positive for both nuclear ZEB1 staining (green) and whole‐cell IL‐6 staining (red); scores = 4. The bottom panel shows a case that was negative for both ZEB1 and IL‐6; scores = 1. The samples were counterstained with DAPI to show cell density in the spot. Original magnification: 20 ×. (C) Kaplan–Meier survival curves of breast and lung cancer patients obtained from a public meta‐analysis database and Kaplan–Meier plotter (Gyorffy *et al*., 2010, 2013). The probability of overall survival of patients as split by median *IL6* and *IL8* expression is shown. Red: *IL6* and *IL8* high expression group; black: *IL6* and *IL8* low expression group.

To evaluate the regulatory functions of ZEB1 in other types of breast cancer cells and cancer tissues, we obtained expression microarray data from the Cancer Cell Line Encyclopedia (CCLE; Barretina *et al*., 2012) and analyzed the relationship of the expression of *ZEB1* with that of inflammatory cytokines, that is, *IL6* and *IL8*, in breast cancer cells. We found that a small subset of the cell lines was ‘double‐positive’ for the expression of *ZEB1* and *IL6* and/or *IL8* (Fig. 5A). In contrast, the expression of *ZEB2* was generally low in breast cancer cells compared to THP‐1 cells, an acute myelogenous leukemia cell line with known ZEB2 function (Li *et al*., 2017), and showed weaker correlation with *IL6* and *IL8* expression than that of ZEB1 (Fig. S3A). We also examined the relationship between ZEB1 and IL‐6 in other types of cancers using a tissue array of 448 cancer tissues from multiple organs and found other double‐positive cancers (Fig. 5B and Fig. S3B,C). These findings suggested that the transcriptional activation of *IL6* by ZEB1 is not restricted to breast cancer cells and tissues and found in a subset of other types of ZEB1‐expressing tumors.

We then examined the prognostic importance of *IL6* and *IL8*, which are regulated by ZEB1 using publically available meta‐analysis database (Kaplan–Meier plotter, http://kmplot.com/analysis/; Fig. 5C). High expression of *IL6* and *IL8* was significantly correlated with poor survival in breast cancer and lung cancer, which is consistent with a previous report (Hartman *et al*., 2013).

### 3.6 ZEB1 promotes HCC1954‐Luc cell proliferation and tumor growth

**Figure 6:**
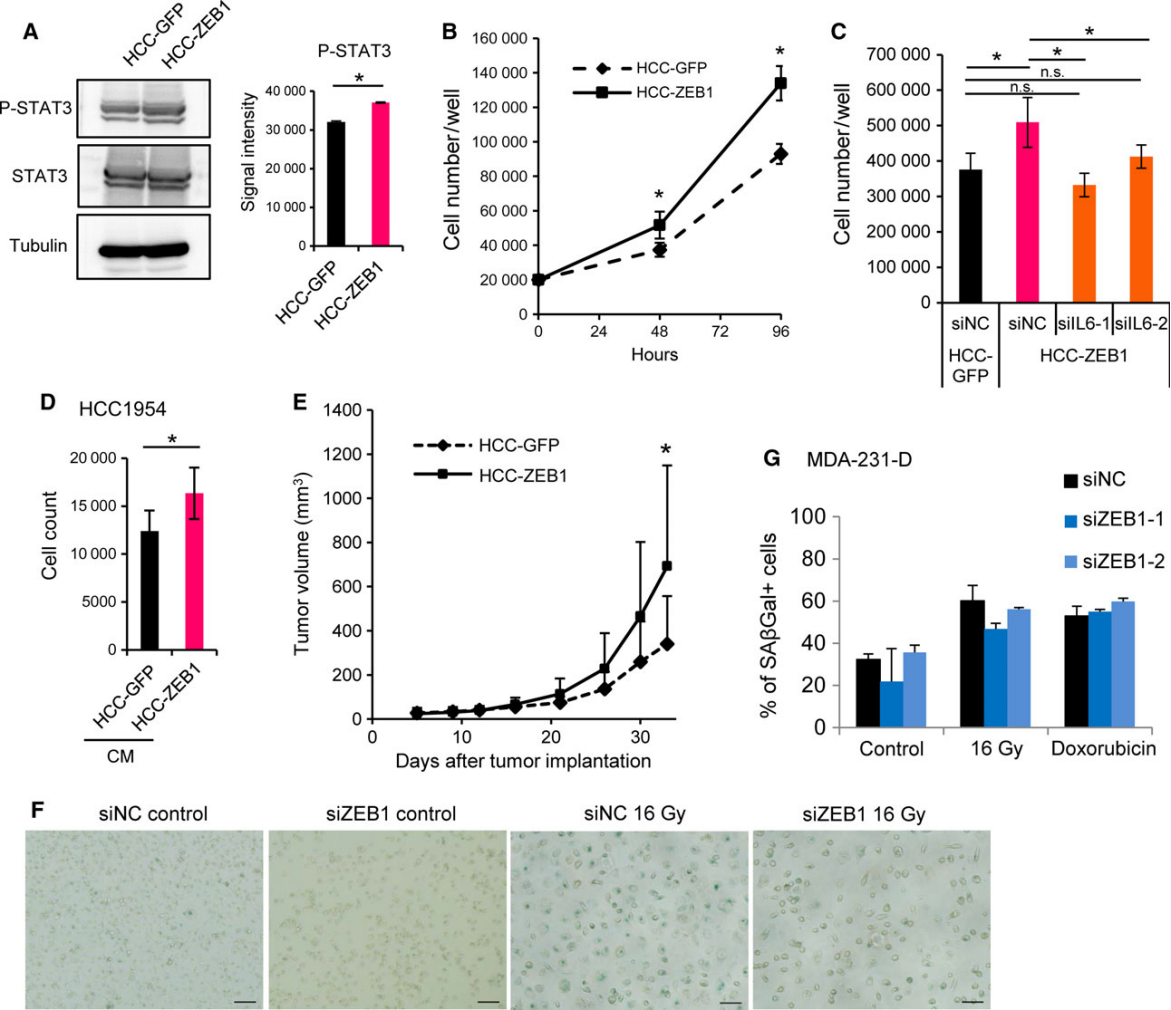
ZEB1 promotes the proliferation and tumor growth of HCC1954‐Luc cells through IL‐6 in an autocrine manner. (A) Tyrosine phosphorylation of STAT3 (P‐STAT3) in control (HCC‐GFP) or ZEB1‐overexpressing (HCC‐ZEB1) HCC1954‐Luc cells. Cell lysates were subjected to immunoblot analysis as indicated. Representative data of two independent experiments are shown. The right panel shows quantification of the P‐STAT3 immunoblot results as the means of two independent experiments. Error bars, S.D.; *P < 0.05. (B) The effect of ZEB1 expression on the number of HCC1954‐Luc cells *in vitro* using the trypan blue exclusion assay. Data are shown as the means of two independent experiments. Error bars, S.D.; *P < 0.05. (C) The effect of IL‐6 siRNA on the number of ZEB1‐overexpressing HCC1954‐Luc cells. Cells were counted 72 h after siRNA transfection. siIL6, IL6 siRNA. Data are shown as the means of two independent experiments. Error bars, S.D., *P < 0.05 by Tukey–Kramer test. n.s.: not significant. (D) HCC1954‐Luc cells were cultured with conditioned medium from control or ZEB1‐overexpressing HCC1954‐Luc cells, and cells were counted after 96 h of incubation. Conditioned media were replenished every 24 h. CM, conditioned medium. Data are shown as the means of two independent experiments. Error bars, S.D.; *P < 0.05. (E) The volumes of tumors formed from control or ZEB1‐overexpressing HCC1954‐Luc cells that were xenografted into nude mice (*n* = 15 for control, *n* = 14 for ZEB1). Error bars, +S.D. *P < 0.05 by the Mann–Whitney *U*‐test. (F, G) Induction of premature cellular senescence was not affected by ZEB1 siRNA. (F) MDA‐231‐D cells were transfected with siRNA as indicated and stained for SA‐βGal 6 days after 16 Gy of irradiation. Three images were taken for each condition, and representative images are shown. Scale bars: 200 μm. (G) MDA‐231‐D cells were transfected with siRNA as indicated and cellular senescence was induced by 16‐Gy irradiation or 75 μm doxorubicin treatment. In terms of irradiation conditions, siRNA was transfected again 2 days after senescence induction. Cells were stained for SA‐βGal 6 days after treatment. Three images were taken for each condition except for one of the controls (two images), and the percentage of SA‐βGal cells was calculated.

Next, we used HCC‐GFP and HCC‐ZEB1 cells to investigate the biological importance of ZEB1‐induced cytokines. Phosphorylation of STAT3 was observed in the absence of IL‐6 expression, suggesting the activation of STAT3 by other pathways, and was moderately enhanced in ZEB1‐expressing HCC1954‐Luc cells (Fig. 6A). ZEB1 promoted the proliferation of HCC1954‐Luc cells (Fig. 6B), and this effect was attenuated by *IL6* siRNA (Fig. 6C). Conditioned medium from HCC‐ZEB1 cell culture enhanced the proliferation of the parental HCC1954‐Luc cells (Fig. 6D). These results suggested that secreted factors, especially IL‐6, contributed to the growth of ZEB1‐expressing HCC‐ZEB1 cells in an autocrine manner. We also found that ZEB1 enhanced tumor growth when HCC1954‐Luc cells were grafted into nude mice (Fig. 6E).

IL‐6, IL‐8, and some other ZEB1‐regulated secretory proteins are related to the senescence‐associated secretory phenotype (Perez‐Mancera *et al*., 2014). However, the induction of cellular senescence in MDA‐231‐D cells by irradiation or doxorubicin treatment, followed by SA‐βGal staining, failed to show a significant correlation between ZEB1 expression and cellular senescence (Fig. 6F and G).

### 3.7 Conditioned medium from ZEB1‐expressing MDA‐231‐D cells increases fibroblast growth in a paracrine manner

**Figure 7:**
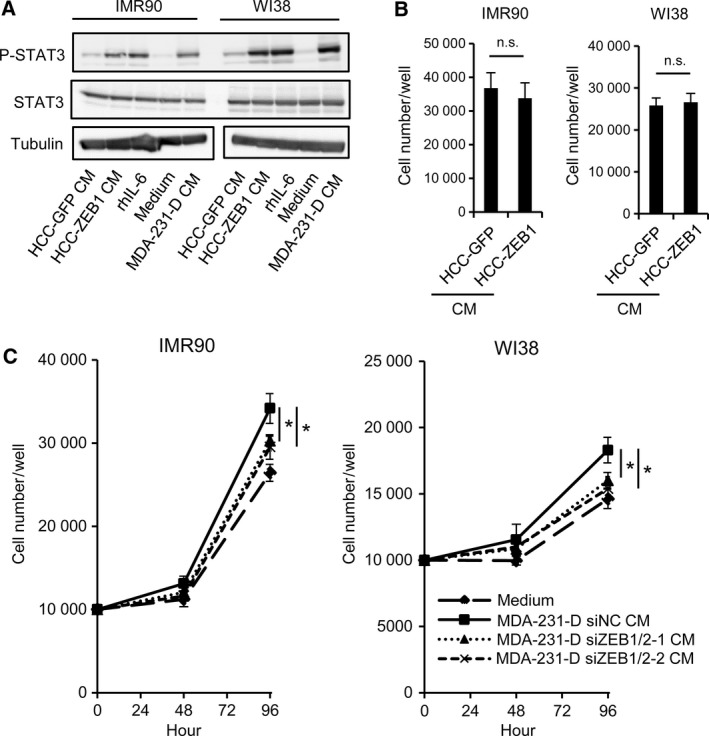
The effect of ZEB1‐regulated secretory factors on fibroblast growth. (A) Tyrosine phosphorylation of STAT3 in IMR90 and WI38 fibroblasts after the addition of conditioned media. Cells were collected 20 min after stimulation, and the cell lysates were subjected to immunoblot analysis as indicated. CM, conditioned medium; rhIL‐6, recombinant human IL‐6 (100 ng·mL^−1^). (B, C) The effect of conditioned medium from cultured breast cancer cells on fibroblast cell growth. The number of IMR90 and WI38 cells was counted after 72 h of incubation using the trypan blue exclusion assay. Conditioned media were obtained from ZEB1‐overexpressing HCC1954‐Luc cells and control cells (HCC‐GFP) (B), or from ZEB1/2‐silenced MDA‐231‐D cells and control cells (C). siZEB1/2‐1, siZEB1‐1 + siZEB2‐1; siZEB1/2‐2, siZEB1‐2 + siZEB2‐2. Conditioned medium was replenished every 24 h. In panel (C), data are shown as the means of three (IMR90) or four (WI38) biological replicates. CM, conditioned medium; n.s., not significant; *P < 0.05.

We further studied the functional relationship between cancer cells and fibroblasts and found that conditioned media from HCC‐ZEB1 and MDA‐231‐D cells upregulated phosphorylated STAT3 in IMR90 and WI38 human fibroblasts (Fig. 7A). Although conditioned medium from HCC‐ZEB1 cells did not increase the growth of IMR90 and WI38 cells (Fig. 7B), conditioned medium from ZEB1/2‐silenced MDA‐231‐D cells significantly decreased the growth of IMR90 and WI38 cells (Fig. 7C). Taken together, these results suggested that ZEB1 expressed in cancer cells contributes to the growth of fibroblasts in paracrine and context‐dependent manners.

### 3.8 ZEB1 induces myeloid cells that express markers characteristic of polymorphonuclear myeloid‐derived suppressor cells (MDSCs) within tumors

**Figure 8:**
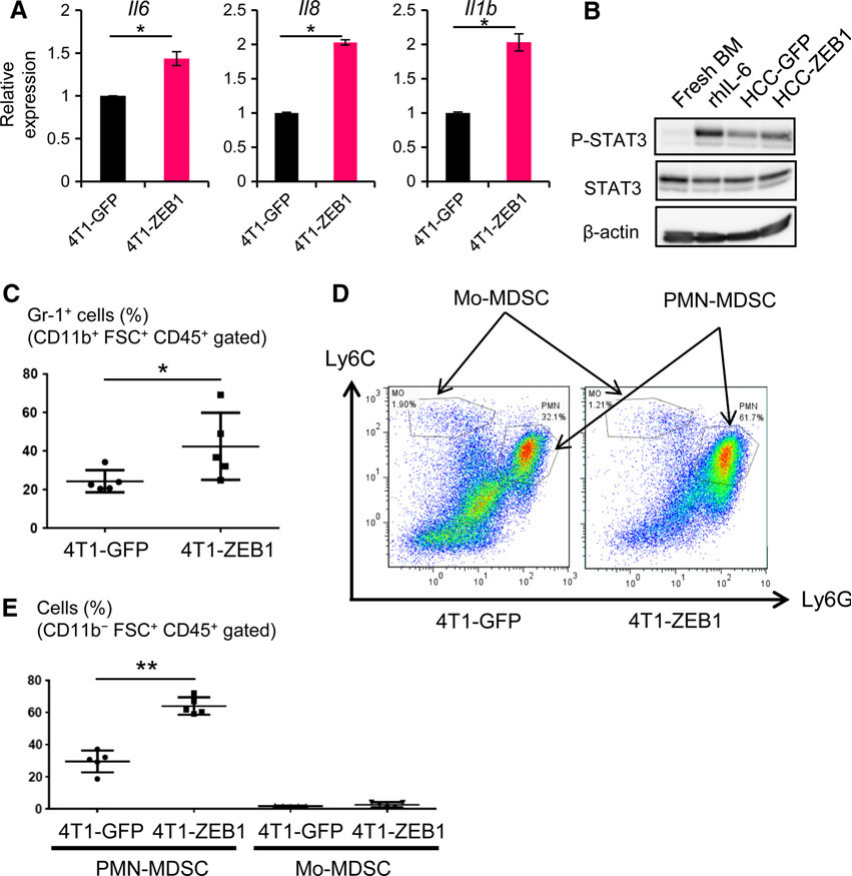
ZEB1‐regulated secretory factors regulate intratumor MDSC populations. (A) qRT‐PCR analysis of the expression of *Il6*,* Il8*, and *Il1b* in ZEB1‐overexpressing mouse breast cancer 4T1 cells. Values were normalized to *Gapdh*. Error bars, S.D. *P < 0.05 by Student's *t*‐test. (B) Tyrosine phosphorylation of STAT3 in mouse BM cells after incubation in conditioned media from control GFP (HCC‐GFP) or ZEB1‐overexpressing (HCC‐ZEB1) HCC1954‐Luc cells for 20 min in cell culture. Cell lysates were subjected to immunoblot analysis as indicated. Recombinant human IL‐6 (rhIL‐6: 40 ng·mL^−1^) was used as a positive control. BM, bone marrow. (C–E) BALB/c mice were injected subcutaneously with ZEB1‐overexpressing 4T1 cells (4T1‐ZEB1). Twenty‐one days after injection, the tumor was removed and analyzed by flow cytometry. 4T1‐GFP, GFP‐expressing control 4T1 cells. (C) Gr‐1‐positive MDSCs (%) in CD11b^+^ FSC ^+^ CD45^+^‐gated cells in tumor tissues (*n* = 5). *P < 0.05 by Mann–Whitney *U*‐test. (D) Quantification of the subpopulation of MDSCs. The gates used to quantify Mo‐MDSCs and PMN‐MDSCs are shown. (E) The percentages of PMN‐MDSCs and Mo‐MDSCs in CD11b^+^ FSC ^+^ CD45^+^‐gated cells. Data are shown as the medians ± S.D. for the five tumors. **P < 0.01 by Mann–Whitney *U*‐test.

Because ZEB1 regulated the production of inflammatory cytokines, including IL‐6, IL‐8, IL‐1β, CXCL1, and CXCL5, we evaluated the paracrine effect of ZEB1‐regulated secretory proteins in terms of their antitumor immune functions. MDSCs comprise heterogeneous cell populations that have the potential for immunosuppressive activity. MDSCs induce immune tolerance in the tumor microenvironment by suppressing cytotoxic T‐lymphocyte activity (Gabrilovich *et al*., 2012; Ugel *et al*., 2015). They are defined as CD11b^+^ Gr‐1^+^ cells in mouse and are categorized as polymorphonuclear (PMN)‐MDSCs and monocytic (Mo)‐MDSCs. PMN‐MDSCs are defined as CD11b^+^ Ly6C^low^ Ly6G^+^ cells that express high levels of arginase 1. In contrast, Mo‐MDSCs are defined as CD11b^+^ Ly6C^high^ Ly6G^−^ cells that express high levels of Nos2 (iNOS; Talmadge and Gabrilovich, 2013). The number of MDSCs in tumor tissue is associated with disease stage in patients with breast cancer (Markowitz *et al*., 2013). To examine the effect of ZEB1‐regulated secretory proteins on the accumulation and maturation of MDSCs *in vivo*, we established murine breast cancer 4T1 cells that overexpressed mouse ZEB1 (4T1‐ZEB1). We confirmed the increased expression of *Il6*,* Il8*, and *Il1b* mRNA in 4T1‐ZEB1 cells versus 4T1‐GFP cells (Fig. 8A).

STAT3 has been reported to be related to the accumulation and expansion of MDSCs (Condamine and Gabrilovich, 2011). To determine whether ZEB1‐regulated secretory proteins affect MDSC development, we focused on STAT3 phosphorylation in mouse bone marrow (BM) cells using conditioned medium from HCC‐ZEB1 cells. We found that STAT3 phosphorylation was enhanced by conditioned medium from HCC‐ZEB1 cells (Fig. 8B).

4T1‐ZEB1 cells were then used in a syngeneic tumor model in immunocompetent BALB/c mice. Tumor size and lung metastasis were not significantly different between 4T1‐ZEB1 cells and 4T1‐GFP cells, possibly because of the highly aggressive nature of 4T1 cells. We then evaluated the accumulation and maturation of MDSCs in the tumor sites by flow cytometry and found that the percentage of Gr‐1‐positive cells was increased in the 4T1‐ZEB1 tumors compared to 4T1‐GFP tumors (Fig. 8C). To characterize the increased MDSCs in more detail, we also evaluated the expression of Ly6C and Ly6G on the cells obtained from tumors to identify PMN‐MDSCs (CD11b^+^ Ly6C^low^ Ly6G^+^) and Mo‐MDSCs (CD11b^+^ Ly6C^high^ Ly6G^−^; Fig. 8D). We found that the percentage of PMN‐MDSCs in the CD11b^+^ FSC^+^ CD45^+^ population of the cells was significantly increased in 4T1‐ZEB1 tumors, while the percentage of Mo‐MDSCs was not (Fig. 8E). Together with the findings from a report showing the immunosuppressive activity of Ly6G^+^ PMN‐MDSCs from 4T1 tumor‐bearing mice (Kim *et al*., 2014), these findings suggested that ZEB1 expression in tumor cells increases the PMN‐MDSC population in CD11b^+^ cells.

## 4 Discussion

Expanding and differential roles of ZEB1 and ZEB2 have been identified in various types of cancers in recent years (Chaffer *et al*., 2013; Krebs *et al*., 2017; Mejlvang *et al*., 2007; Morel *et al*., 2017; Si *et al*., 2015; Spaderna *et al*., 2008). ZEB1 is expressed primarily in nonepithelial cells, and a genome‐wide analysis of ZEB1‐binding regions performed in an adipogenesis model clearly shows its physiological importance (Gubelmann *et al*., 2014). Therefore, it is not surprising that the main consequence of the ectopic expression of ZEB1 differs greatly between cancer cell types. This is because transcription factor binding regions are affected by the epigenetic environment in the cells, which is dynamically regulated by TGF‐β and other extracellular stimuli (Ostuni *et al*., 2013). The observation that ZEB1 has differential effects on the expression of genes that are either downregulated or upregulated by TGF‐β in MDA‐231‐D cells also supports the importance of cellular context. In addition, Hs578T and Panc‐1 cells showed very similar ZEB1 binding profile, which likely reflected both similar binding strength and regulatory mechanism of ZEB1 binding.

Thus, apart from its general role as an EMT inducer, ZEB1 and ZEB2 may have multiple functions that will be elucidated by analyses in specific cancer types in the future.

Inflammatory cytokines play crucial roles in various aspects of cancer, including cancer development, progression, treatment resistance, and prognosis. IL‐1β promotes colon cancer cell stemness and invasiveness (Li *et al*., 2012). IL‐8 is also associated with cancer stem cell‐like properties, and its expression correlates with poor prognosis in human pancreatic cancer (Chen *et al*., 2014). Of these inflammatory cytokines, IL‐6 is reported to play especially important roles in the development of lung and breast cancers (Gao *et al*., 2007; Sansone *et al*., 2007). With regard to the effect of these cytokines on the EMT, previous reports focused mainly on the regulation of EMT‐related downstream factors, with ZEB1 reported as a target gene of certain inflammatory cytokines (Liu *et al*., 2015; Peinado *et al*., 2007). A recent report suggested that inflammation induces disseminated, dormant cancer cells to form metastatic tumors through functions of ZEB1 (De Cock *et al*., 2016). It is also reported that ZEB2 is induced by inflammation (Katoh and Katoh, 2009). However, the induction of IL‐6 and IL‐8 by ZEB1 and ZEB2, an inverse relationship that was revealed in this study, has not been investigated in detail.

It is widely accepted that cancer cells show EMT‐like phenotypes due to the production of various cytokines. For example, elevated expression of *IL6* and other chemoresistance‐related genes accompanies the EMT in a mouse breast cancer model (Fischer *et al*., 2015). Our finding that ZEB1 and ZEB2 are inducers of inflammatory cytokines is supported by a report by Suarez‐Carmona *et al*. (2015) that Slug, Snail, and other EMT‐related transcription factors regulate the production of soluble factors, such as IL‐8, IL‐6, sICAM‐1, PAI‐1, and GM‐CSF/CSF2. Taken together, these data indicate that inflammatory cytokines induce EMT‐related transcription factors and vice versa in certain cancer cells to enhance tumor progression. These processes can be targeted by molecular therapies.

Our findings indicated that ZEB1 and partly ZEB2 regulated the characteristic inflammatory phenotype of breast cancer cells, in part through IL‐6 and IL‐8. The ZEB1‐regulated inflammatory phenotype identified in this study was characterized by enhanced breast cancer cell growth, fibroblast proliferation, and PMN‐MDSC accumulation, although each of these was observed only in certain cell types and was context dependent. The correlation between IL‐6 production and cancer proliferation has been reported in various types of cancers, including lung, prostate, and breast cancers (Gao *et al*., 2007; Giri *et al*., 2001; Sansone *et al*., 2007; Yamaji *et al*., 2004). IL‐6 secreted by cancer cells activates STAT3, and induces downstream events, including cancer cell proliferation and apoptosis inhibition. Our results support these reports and revealed that IL‐6, as a central soluble factor that was positively regulated by ZEB1, constantly activated STAT3 in HCC1954‐Luc cells and exhibited cell‐proliferating potency both in cell culture and *in vivo*. IL‐6, which was regulated by ZEB1 and ZEB2 in breast cancer cells, also induced the proliferation of fibroblasts in a context‐dependent manner, suggesting that ZEB1 and ZEB2 regulate the production and function of cancer‐associated fibroblasts, which are the main constituents of tumor microenvironments, thereby enhancing tumor growth (Kalluri and Zeisberg, 2006; Xing *et al*., 2010).

In terms of MDSCs, IL‐6 strongly induces their accumulation and maturation. IL‐6 is reported to restore the impaired accumulation of MDSCs and tumor progression in tumor‐bearing mice lacking IL‐1β or indoleamine 2,3‐dioxygenase (IDO; Bunt *et al*., 2007; Smith *et al*., 2012). Activated STAT3 is the main regulator of IL‐6 in MDSCs, inducing cell survival, cell proliferation (Xin *et al*., 2009), and immunosuppressive activity (Kujawski *et al*., 2008). IL‐8 also enhances PMN‐MDSC infiltration into tumor tissues (Kumar *et al*., 2016; Sandhu *et al*., 2000). Furthermore, IL‐1β is reported to induce the accumulation of MDSCs (Bunt *et al*., 2007; Elkabets *et al*., 2010). In addition to IL‐6, IL‐8, and IL‐1β, another ZEB1‐regulated cytokine, G‐CSF/CSF3, also enhances the accumulation of MDSCs (Talmadge and Gabrilovich, 2013). ZEB1 also regulates the expression of the chemokines CXCL1 and CXCL5, which are the ligands for the CXCR2 receptor, and increases the infiltration of PMN‐MDSCs (Acharyya *et al*., 2012; Katoh *et al*., 2013; Toh *et al*., 2011). Although the present study showed no additional effect of ZEB1 on tumor progression or on the metastasis of 4T1 cells, which may be due to the aggressive nature of the parental 4T1 cells, these observations suggest that inflammatory cytokines induced by ZEB1 play critical roles in the progression of cancer in a context‐dependent manner.

In basal‐type breast cancer cells, high ZEB1 expression was observed even in the absence of TGF‐β (Horiguchi *et al*., 2012). Of note, in mammary carcinomas that do not express the TGF‐β type II receptor, CXCL5 expression is increased, resulting in the recruitment of MDSCs (Yang *et al*., 2008). Thus, although TGF‐β induces the expression of ZEB1 and ZEB2, which function as key transcription factors in the induction of the EMT in various types of epithelial cells, TGF‐β signaling and ZEB1 and ZEB2 act in opposite ways in cancer cells in some contexts, including in the regulation of MDSCs, and they play distinct roles in cancer progression.

In conclusion, ZEB1 and ZEB2, through the induction of various cytokines, including IL‐6 and IL‐8, facilitate tumor growth both in autocrine and in paracrine manners in basal‐type breast cancer cells. Future studies will focus on evaluating these extracellular proteins as potential anticancer targets to inhibit the progression of cancer.

## Availability of data and materials

The raw ChIP‐seq and RNA‐seq data are available at GEO (GSE89206).

## Author contributions

AK and YT performed most of the *in vitro* experiments, together with SH, MH, JN, YY, and SE, AK, YT, TS, and KT performed the *in vivo* experiments. AK, YT, and YM performed the immunohistochemistry. AM and DK determined the experimental conditions for the ZEB1 ChIP‐seq analysis. YT, AK, DK, and MM acquired and analyzed the high‐throughput sequencing data. DK and KM designed experiments and analyzed data. TM established the HCC1954‐Luc cells. YT, AK, MM, KM, and DK wrote the manuscript.

## Supporting information


**Fig. S1.** Specificity of ZEB1 and ZEB2 antibodies and the result of GSEA analysis showing the effect of ZEB1 or ZEB2 siRNA in MDA‐231‐D cells.
**Fig. S2.** The effect of ZEB1/2 siRNA on the expression of inflammatory response genes.
**Fig. S3.** Tissue array analysis of ZEB1 and IL‐6 expression using fluorescent immunohistochemistry.
**Fig. S4.** Efficiency of IL6 siRNA and the amount of ZEB1 protein in 4T1 breast cancer cells.

**Table S1.** Sequences of the primers used for RT‐PCR.

**Table S2.** A list of ZEB1‐bound inflammatory response genes.
