# Supplementary material for: ZEB1‐regulated inflammatory phenotype in breast cancer cells
Source: Mol Oncol. 2017 Jul 11;11(9):1241–62. doi: 10.1002/1878-0261.12098 (PMC5579340; doi:10.1002/1878-0261.12098)

Supplementary Figure S1

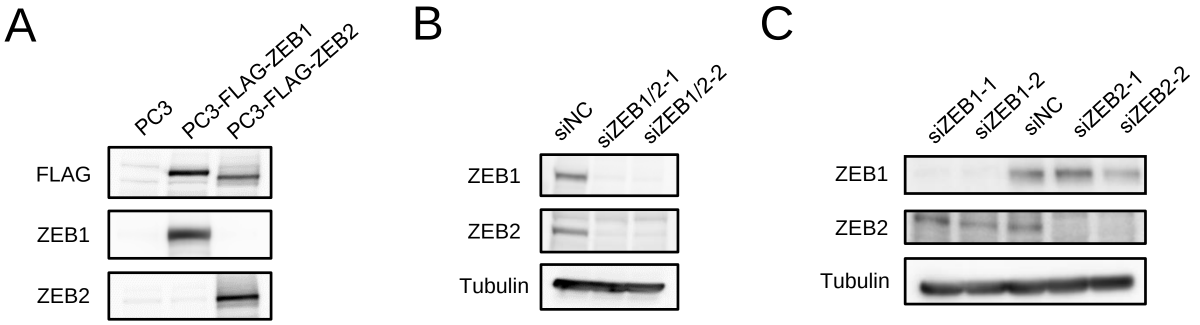

D Result of GSEA: hallmarks

ZEB1 as activators

siZEB1-1

NAME	NES
HALLMARK_INFLAMMATORY_RESPONSE	1.62
HALLMARK_IL6_JAK_STAT3_SIGNALING	1.57

siZEB1-2

NAME	NES
HALLMARK_E2F_TARGETS	2.38
HALLMARK_G2M_CHECKPOINT	2.37
HALLMARK_MYC_TARGETS_V1	2.12
INFLAMMATORY_RESPONSE	1.91
HALLMARK_MTORC1_SIGNALING	1.90
HALLMARK_MYC_TARGETS_V2	1.86
HALLMARK_MITOTIC_SPINDLE	1.73
HALLMARK_UNFOLDED_PROTEIN_RESPONSE	1.66
HALLMARK_ALLOGRAFT_REJECTION	1.52

ZEB2 as activators

siZEB2-1

NAME	NES
HALLMARK_INFLAMMATORY_RESPONSE	2.17
HALLMARK_KRAS_SIGNALING_UP	2.16
HALLMARK_INTERFERON_ALPHA_RESPONSE	2.11
HALLMARK_COAGULATION	1.99
HALLMARK_INTERFERON_GAMMA_RESPONSE	1.92
HALLMARK_TNFA_SIGNALING_VIA_NFKB	1.90
HALLMARK_EPITHELIAL_MESENCHYMAL_TRANSITION	1.83
HALLMARK_ANGIOGENESIS	1.82
HALLMARK_COMPLEMENT	1.68
HALLMARK_ALLOGRAFT_REJECTION	1.66
HALLMARK_IL6_JAK_STAT3_SIGNALING	1.63

siZEB2-2

NAME	NES
HALLMARK_KRAS_SIGNALING_UP	1.70
...	...
HALLMARK_INFLAMMATORY_RESPONSE	1.34

Supplementary Figure S2

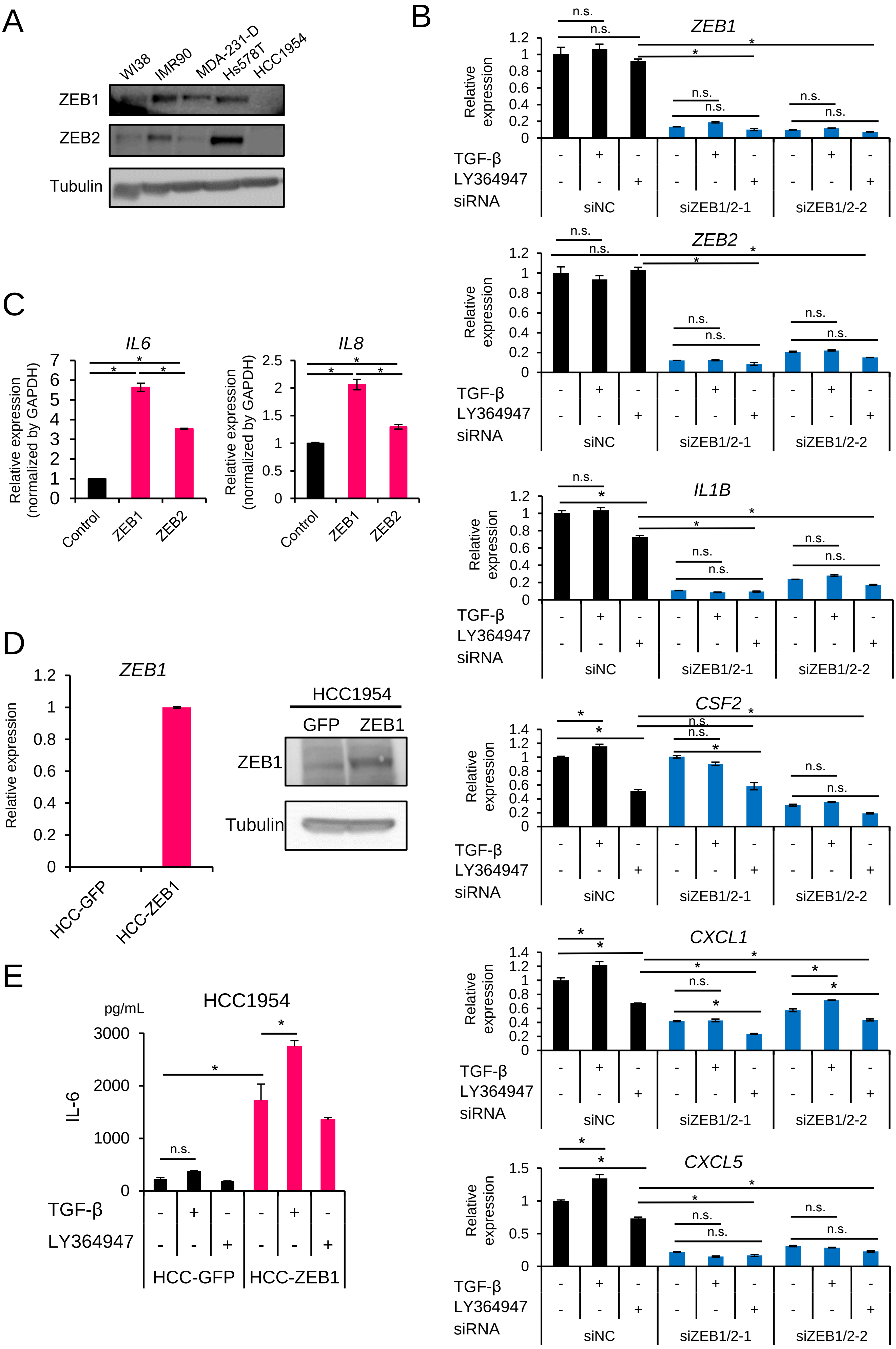

Supplementary Figure S3

A

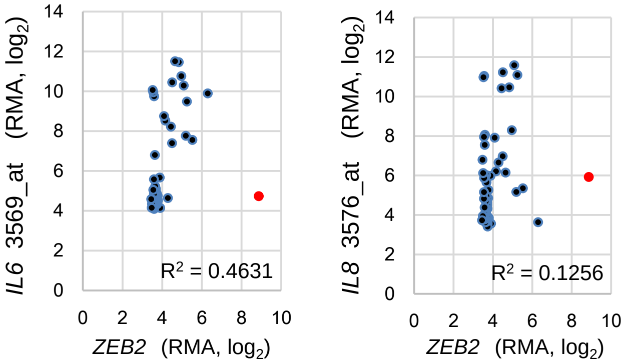

B

IL-6 / Score	1	2	3	4
4	0	2	17	56
3	30	81	89	21
2	47	56	14	3
1	29	2	1	0

ZEB1

Tubulin / Score	1	2	3	4
4	3	6	12	9
3	7	10	17	34
2	18	31	43	23
1	72	90	46	12

ZEB1

C

Bladder

IL-6 / Score	1	2	3	4
4	0	0	0	0
3	1	1	4	1
2	5	1	0	0
1	6	0	0	0

ZEB1

Esophagus

IL-6 / Score	1	2	3	4
4	0	0	0	2
3	2	8	2	0
2	0	3	3	0
1	0	0	0	0

ZEB1

Lung

IL-6 / Score	1	2	3	4
4	0	0	0	8
3	5	11	8	2
2	4	5	4	1
1	0	0	0	0

ZEB1

Skin（Sq）

IL-6 / Score	1	2	3	4
4	0	0	2	1
3	3	4	3	1
2	3	3	0	0
1	0	0	0	0

ZEB1

Uterus

IL-6 / Score	1	2	3	4
4	0	0	0	0
3	1	3	5	0
2	5	3	2	1
1	0	0	0	0

ZEB1

Cerebrum

IL-6 / Score	1	2	3	4
4	0	0	0	2
3	0	2	6	1
2	0	4	2	0
1	1	1	1	0

ZEB1

Head and neck

IL-6 / Score	1	2	3	4
4	0	0	1	1
3	3	7	2	0
2	1	2	2	0
1	1	0	0	0

ZEB1

Lymph node

IL-6 / Score	1	2	3	4
4	0	0	1	8
3	0	2	2	5
2	0	2	0	0
1	0	0	0	0

ZEB1

Skin（melanoma）

IL-6 / Score	1	2	3	4
4	0	0	0	0
3	2	6	7	2
2	1	2	0	0
1	0	0	0	0

ZEB1

Breast

IL-6 / Score	1	2	3	4
4	0	0	0	0
3	2	2	5	0
2	5	1	0	0
1	5	0	0	0

ZEB1

Kidney

IL-6 / Score	1	2	3	4
4	0	0	4	7
3	1	2	1	1
2	1	2	0	1
1	0	0	0	0

ZEB1

Ovary

IL-6 / Score	1	2	3	4
4	0	0	0	0
3	2	2	5	1
2	2	3	0	0
1	5	0	0	0

ZEB1

Stomach

IL-6 / Score	1	2	3	4
4	0	0	3	0
3	0	3	10	0
2	1	1	0	0
1	2	0	0	0

ZEB1

Colon

IL-6 / Score	1	2	3	4
4	0	0	0	0
3	2	3	5	0
2	4	5	0	0
1	1	0	0	0

ZEB1

Liver

IL-6 / Score	1	2	3	4
4	0	1	1	9
3	1	3	2	3
2	0	0	0	0
1	0	0	0	0

ZEB1

Pancreas

IL-6 / Score	1	2	3	4
4	0	0	0	0
3	2	2	7	0
2	3	3	0	0
1	3	0	0	0

ZEB1

Testis

IL-6 / Score	1	2	3	4
4	0	0	1	1
3	0	5	3	2
2	4	3	1	0
1	0	0	0	0

ZEB1

Prostate

IL-6 / Score	1	2	3	4
4	0	0	0	1
3	1	5	4	2
2	1	5	0	0
1	1	0	0	0

ZEB1

Soft tissue

IL-6 / Score	1	2	3	4
4	0	1	0	3
3	0	4	3	0
2	3	1	0	0
1	4	1	0	0

ZEB1

Thyroid gland

IL-6 / Score	1	2	3	4
4	0	0	4	12
3	0	0	4	0
2	0	0	0	0
1	0	0	0	0

ZEB1

Uterine cervix

IL-6 / Score	1	2	3	4
4	0	0	0	1
3	1	6	1	0
2	4	7	0	0
1	0	0	0	0

ZEB1

D

Bladder

Tubulin / Score	1	2	3	4
4	0	0	2	0
3	1	0	2	0
2	8	1	1	0
1	4	1	0	0

ZEB1

Esophagus

Tubulin / Score	1	2	3	4
4	0	0	0	0
3	0	0	0	1
2	0	0	1	0
1	2	11	4	1

ZEB1

Lung

Tubulin / Score	1	2	3	4
4	1	0	1	1
3	0	3	1	6
2	2	5	4	2
1	6	8	6	2

ZEB1

Skin（Sq）

Tubulin / Score	1	2	3	4
4	0	1	1	0
3	0	1	0	1
2	0	1	3	1
1	6	2	1	0

ZEB1

Uterus

Tubulin / Score	1	2	3	4
4	0	1	2	0
3	3	1	1	0
2	0	3	3	0
1	3	1	1	1

ZEB1

Cerebrum

Tubulin / Score	1	2	3	4
4	0	0	0	0
3	0	1	4	2
2	0	3	4	1
1	1	3	0	0

ZEB1

Head and neck

Tubulin / Score	1	2	3	4
4	0	1	0	0
3	0	0	0	1
2	0	1	2	0
1	5	7	3	0

ZEB1

Lymph node

Tubulin / Score	1	2	3	4
4	0	1	2	0
3	0	0	0	8
2	0	2	0	5
1	0	1	0	0

ZEB1

Skin（melanoma）

Tubulin / Score	1	2	3	4
4	1	1	1	0
3	0	0	2	0
2	1	3	1	2
1	1	4	2	0

ZEB1

Breast

Tubulin / Score	1	2	3	4
4	0	0	1	0
3	0	1	0	0
2	3	0	2	0
1	9	2	2	0

ZEB1

Kidney

Tubulin / Score	1	2	3	4
4	0	0	0	3
3	0	0	0	1
2	0	0	4	1
1	2	4	1	4

ZEB1

Ovary

Tubulin / Score	1	2	3	4
4	1	0	0	0
3	1	1	1	0
2	0	1	1	0
1	7	3	3	1

ZEB1

Stomach

Tubulin / Score	1	2	3	4
4	0	0	0	0
3	1	0	0	0
2	1	0	4	0
1	1	4	8	0

ZEB1

Colon

Tubulin / Score	1	2	3	4
4	0	0	0	0
3	0	0	0	0
2	0	4	3	0
1	4	4	2	0

ZEB1

Liver

Tubulin / Score	1	2	3	4
4	0	0	0	4
3	0	0	0	3
2	0	3	1	4
1	1	1	2	1

ZEB1

Pancreas

Tubulin / Score	1	2	3	4
4	0	0	0	0
3	0	0	2	0
2	3	1	1	0
1	5	4	4	0

ZEB1

Testis

Tubulin / Score	1	2	3	4
4	0	0	0	0
3	0	0	0	0
2	0	1	4	1
1	4	7	1	2

ZEB1

Prostate

Tubulin / Score	1	2	3	4
4	0	0	1	0
3	1	0	1	2
2	0	1	1	1
1	2	9	1	0

ZEB1

Soft tissue

Tubulin / Score	1	2	3	4
4	0	1	1	0
3	0	2	0	1
2	0	0	0	1
1	4	2	2	0

ZEB1

Thyroid gland

Tubulin / Score	1	2	3	4
4	0	0	0	1
3	0	0	3	7
2	0	0	2	4
1	0	0	3	0

ZEB1

Uterine cervix

Tubulin / Score	1	2	3	4
4	0	0	0	0
3	0	0	0	1
2	0	1	1	0
1	5	12	0	0

ZEB1

Supplementary Figure S4

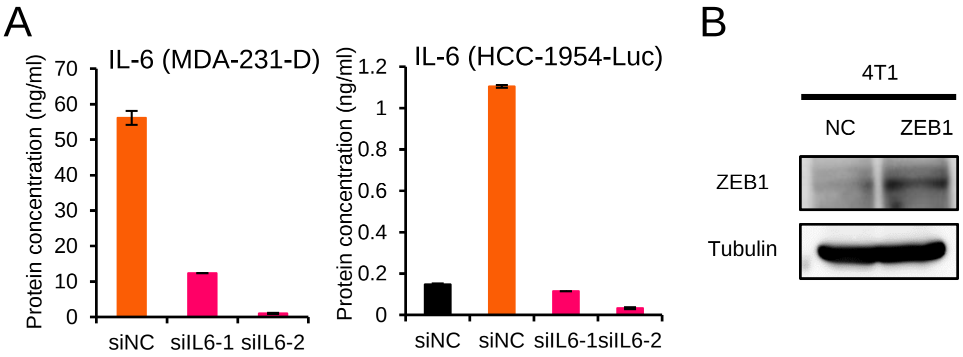

Supplement: Supplementary file 1 — Fig. S1. Specificity of ZEB1 and ZEB2 antibodies and the result of GSEA analysis showing the effect of ZEB1 or ZEB2 siRNA in MDA‐231‐D cells. Fig. S2. The effect of ZEB1/2 siRNA on the expression of inflammatory response genes. Fig. S3. Tissue array analysis of ZEB1 and IL‐6 expression using fluorescent immunohistochemistry. Fig. S4. Efficiency of IL6 siRNA and the amount of ZEB1 protein in 4T1 breast cancer cells. [file MOL2-11-1241-s001.pdf]
