# Supplementary material for: ZEB1‐regulated inflammatory phenotype in breast cancer cells
Source: Mol Oncol. 2017 Jul 11;11(9):1241–62. doi: 10.1002/1878-0261.12098 (PMC5579340; doi:10.1002/1878-0261.12098)
Supplement: Supplementary file 2 — Table S1. Sequences of the primers used for RT‐PCR. [file MOL2-11-1241-s002.docx]

**SUPPLEMENTAL MATERIALS**

**Table S1. Sequences of the primers used for RT-PCR.**

| Human | *ZEB1* | fwd | CAATGATCAGCCTCAATCTGCA |
| --- | --- | --- | --- |
|  |  | rev | CCATTGGTGGTTGATCCCA |
|  | *ZEB2* | fwd | TGCTAACCCAAGGAGCAGGTAA |
|  |  | rev | CCACTGTGAATTCGCAGGTGT |
|  | *IL6* | fwd | CACACAGACAGCCACTCACC |
|  |  | rev | TTTTCTGCCAGTGCCTCTTT |
|  | *IL8* | fwd | GTGCAGTTTTGCCAAGGAGT |
|  |  | rev | AATTTCTGTGTTGGCGCAGT |
|  | *IL1B* | fwd | GGACAAGCTGAGGAAGATGC |
|  |  | rev | TCGTTATCCCATGTGTCGAA |
|  | *CSF2* (GMCSF) | fwd | TTCTGCTTGTCATCCCCTTT |
|  |  | rev | CTTGGTCCCTCCAAGATGAC |
|  | *GAPDH* | fwd | GAAGGTGAAGGTCGGAGTC |
|  |  | rev | GAAGATGGTGATGGGATTTC |
|  | *CXCL1* | fwd | GAAAGCTTGCCTCAATCCTG |
|  |  | rev | CACCAGTGAGCTTCCTCCTC |
|  | *CXCL5* | fwd | TCTGCAAGTGTTCGCCATAG |
|  |  | rev | TTGTTTCCACCGTCCAAAAT |
| Mouse | *Zeb1* | fwd | TTCTGCAGCAACAAGACACC |
|  |  | rev | TCATCATGACTGCTGGCTTC |
|  | *Zeb2* | fwd | CGACACGGCCATTATTTACC |
|  |  | rev | GGCAAAAGCATCTGGAGTTC |
|  | *Gapdh* | fwd | TGCAGTGGCAAAGTGGAGATT |
|  |  | rev | TGCCGTTGAATTTGCCGT |
|  | *Il6* | fwd | CCGGAGAGGAGACTTCACAG |
|  |  | rev | TCCACGATTTCCCAGAGAAC |
|  | *Il8* | fwd | ATGTGGATGGGAACAACGAT |
|  |  | rev | GTCAGAACGTGGCGGTATCT |
|  | *Il1b* | fwd | CCCAAGCAATACCCAAAGAA |
|  |  | rev | GCTTGTGCTCTGCTTGTGAG |

fwd, forward; rev, reverse.

**SUPPLEMENTARY FIGURE LEGENDS**

**Figure S1. Specificity of ZEB1 and ZEB2 antibodies and the result of GSEA analysis showing the effect of ZEB1 or ZEB2 siRNAs in MDA-231-D cells.**

(A) Specificity of ZEB1 and ZEB2 antibodies. HEK293T cells were collected 48 hours after transfection and immunoblotting was performed using the antibodies as indicated.

(B) Efficiency of ZEB1 and ZEB2 siRNAs determined by immunoblotting in MDA-231-D cells.

(C) The effect of siRNAs for ZEB1 or ZEB2 detemined by immunoblotting in MDA-231-D cells.

(D) Downregulated gene sets in MDA-231-D cells that were differentially expressed after treatment with ZEB1 or ZEB2 siRNAs. The most enriched MSigDB hallmark signatures that had normalized enrichment scores (NES) >1.5 were listed. For the data obtained from siZEB2-2 transfected sample, NES score of HALLMARK_INFLAMMATORY_RESPONSE was additionally shown.

**Figure S2. The effect of ZEB1/2 siRNAs on the expression of inflammatory response genes.**

(A) The amount of endogenous ZEB1 and ZEB2 proteins in basal type breast cancer cells used in this study. WI38 and IMR90 were used as positive controls.

(B) The effect of siRNAs targeting ZEB1/2 on the expression of *ZEB1* and *ZEB2* and inflammatory response genes treated with TGF-β or LY364947. Samples obtained from MDA-231-D cells that were used in the experiments shown in Figure 3B were used for this evaluation. Data are shown as the means of two biological replicates. Error bars, S.D. *p<0.05, n.s., not significant.

(C) qRT-PCR analysis of *IL6* and *IL8* expression 48 hours after transfection of ZEB1- or ZEB2- expressing vector in HCC1954-Luc cells. Error bars, S.D. *p<0.05

(D) The expression level of exogenous ZEB1 in HCC1954-Luc cells was determined by qRT-PCR using mouse ZEB1-specific primers and by immunoblotting.

(E) The amount of secreted IL6 protein in ZEB1 expressing HCC-1954-Luc cells treated with TGF-β or LY364947 determined by ELISA. Error bars, S.D. *p<0.05

**Figure S3. Tissue array analysis of ZEB1 and IL-6 expression using fluorescent immunohistochemistry.**

(A) Correlation analysis of the expression of *ZEB2* and cytokines in breast cancer cell lines using data obtained from the Cancer Cell Line Encyclopedia (CCLE). Breast cancer cell lines were selected for the analysis, and each dot represents a cell line, as shown in Figure 5A. Affymetrix microarray probe IDs for *IL6* and *IL8* are shown after the gene symbols on the y-axis. RMA, robust multi-array average. A red dot represents the gene expression in THP-1 cells as a positive control of ZEB2 expression.

(B and C) Score tables showing the amount of ZEB1 and IL-6 proteins in tissue array samples. The immunofluorescent signal intensity of nuclear ZEB1 and cellular IL-6 in each sample was scored from 1 (low intensity) to 4 (high intensity). A summary of the data from all samples was shown in (B).

(D) Anti-tubulin staining and scoring was performed as a control.

**Figure S4. Efficiency of IL6 siRNAs and the amount of ZEB1 protein in 4T1 breast cancer cells.**

(A) Knockdown efficiency of the siRNAs against *IL6* in MDA-231-D and HCC1954 cells determined by ELISA. Error bars, S.D.

(B) The amount of ZEB1 expression in control and ZEB1-expressing mouse breast cancer 4T1 cells determined by immunoblotting.
